# Supportive Relationships in Autism Spectrum Disorder: Perspectives of Individuals with ASD and Supporters

**DOI:** 10.3390/bs6040023

**Published:** 2016-11-03

**Authors:** Jodi Robledo, Anne M. Donnellan

**Affiliations:** 1 School of Education, California State University San Marcos, San Marcos, CA 92096, USA; 2 School of Leadership and Education Sciences, University of San Diego, San Diego, CA 92110-2492, USA; anned@sandiego.edu

**Keywords:** Autism Spectrum Disorder, supportive relationships, trust, unity, support, intimacy, mutuality, reciprocity

## Abstract

This study explored 17 dyads of academically successful people with Autism Spectrum Disorder (ASD) and individuals who they identified as supportive. Qualitative methods, including in-depth interviews, participant observations, and document analysis, were used to study these supportive relationships. The purpose of the study was to develop a substantive grounded theory regarding supportive relationships within the lives of individuals with ASD. A dynamic model of supportive relationships emerged, with trust, unity, and support as the three core categories of these relationships. The data suggest that the quality of the relationship between an individual with ASD and the support provider can be a critical factor within effective support. These findings suggest that there is much yet to be learned about the social world of individuals with ASD.

## 1. Introduction

In 1943, child psychiatrist Leo Kanner was the first to describe the condition that would later be called autism. Through his observations of 11 children, Kanner noted behavioral features that distinguished this group from typically developing peers and other childhood disorders such as childhood schizophrenia or child psychosis. Kanner [1] described these children as having a disturbance of affective development that resulted in a profoundly disturbed pattern of social development. Around the same time Kanner was making his observations, Hans Asperger, an Austrian psychiatrist, described a set of behavioral features that were similar to Kanner’s account [2]. Asperger also felt that atypical social development was at the core of this syndrome. As he stated: “The autist is only himself and is not an active member of a greater organism which he is influenced by and which he influences constantly” [2] (p. 38).

Challenges with social interactions, social behavior, and social understanding remain the defining characteristics of Autism Spectrum Disorder (ASD). Recent updates to the fifth edition of the Diagnostic and Statistical Manual of Mental Disorders [3] continue to include impairment in social development, interaction, and relationships as hallmark features of the disorder. The social characteristics of ASD have been well documented through empirical studies (see [4] for a review). In summary, studies conclude that the social deficits considered hallmark to ASD include: lack of cooperative play, deficits in joint attention and eye gaze, lack of empathetic expression and shared enjoyment, lack of reciprocity in social interactions, and lack of coordination of social behaviors that signal social intention. Researchers in ASD traditionally approach social behavior, interactions, and relationships from a positivist-reduction perspective. Typically, social interactions are studied by looking at discrete social behaviors outside of the context of real-life relationships. Most often these behaviors are studied through sociometric techniques and clinical observations that take place in settings outside of the individual’s natural environment. While this literature describes in a general way the deficits associated with the disorder, it does not provide a rich description of how these challenges affect the everyday life experiences of those with ASD or the experiences of others who interact with them.

In the last 20 years there has been an explosion of published first-hand accounts from individuals with the ASD label that begin to provide a description of how these social challenges affect their day-to-day lives [5,6,7,8,9,10,11,12,13] and first-hand accounts used in research studies [14,15,16,17]. Although it is possible that these individuals with ASD are a select and non-representative group, it is undeniable that they have much to teach us about the world of ASD. Most importantly, these first-hand accounts brought the perspective of the labeled individual into the conversation for the first time.

Numerous individuals with ASD have reported, either through published first-hand accounts or at professional conferences, that significant people in their lives, such as parents, siblings, friends, teachers, and paid support staff, have provided them immense support. These brief accounts are really all we know about these relationships.

“Best practices” in the education and support of people with ASD focuses on formal supports in the form of comprehensive programs based on professional interventions. However, these programs primarily focus on teaching specific skills or decreasing, managing, or modifying inappropriate behaviors. With only a few exceptions, these programs do not promote the development and maintenance of personal relationships; in fact, they may even hinder them [18]. Instead, personal relationships, especially friendships, are viewed as something to explore only after individuals have reached some specific skill level or level of independence [19] and even then, relationships are viewed as leisure activities, not as sources of support and growth. However, recent empirical studies have suggested that the quality of the relationships between the individual with a disability, including ASD, and the people who support them might be the most critical element of successful intervention, treatment, and education [20,21].

Although first-hand accounts of individuals with ASD have provided us brief descriptions of supportive relationships in their lives, there is much more to know about these relationships. We know little about what these relationships look like and how they provide support for individuals with ASD. In fact, this topic has rarely been explored with individuals with any type of disability. When one conducts an academic search using the words “support” and “disability”, numerous research reports surface that describe support for everyone but the person with a disability. A multitude of literature is available on supporting parents, siblings, teachers, and paid support staff, yet little research focuses on supporting the person with a disability.

The present study is an effort to fill the gap in existing knowledge and to provide a rich description of these types of supportive relationships. We explored, through qualitative methods, relationships that individuals with ASD identified as supportive. The aim of this study was to describe and understand the experiences and perspectives of both people with ASD and significant individuals who have supported them.

The study has been designed as an example of what Bogdan and Taylor [22] described as “optimistic research”, which focuses on highlighting positive examples with a view towards change. In this study, positive examples include individuals with ASD who have been defined as “academically successful”, which will be discussed in greater detail later. Bogdan and Taylor argued that the field of special education already has research that focuses on the “dark side”. This type of research is often hard to take into practice because it provides little guidance. Instead, it points out what we should not do, providing few examples of positive practice. Optimistic research aims to be both positive about practice and helpful to practitioners. By focusing on individuals with ASD who have been defined as “academically successful”, we may get a better picture of how successful individuals with ASD are supported. In turn, this may provide guidance for how we should provide support to all individuals with ASD and revise our understanding of the nature of ASD.

The purpose of this study was to develop a substantive grounded theory about supportive relationships for people with autism. This theory was developed through the use of grounded theory [23,24], more specifically constructivist grounded theory [25,26,27]. The theory was developed through analysis of the data that emerged during the study. The ultimate goal was to create a substantive theory that can be taken directly into practice.

Additional purposes of this study include: (1) documenting the experiences of individuals with ASD who are “academically successful” and exploring aspects of their experiences with social support that have enhanced or limited their experiences; (2) exploring whether and how the mode of communication influences the quality of the supportive relationship; and (3) exploring the qualities and dimensions of the relationships. This study will also deepen our understanding of the capacities of people with ASD to engage in social relationships.

The research questions below allowed for a rich qualitative description of the relationships from each person’s perspective and for the emergence of a substantive grounded theory.

The questions that guided the study included:

(1) How do individuals with ASD and the people who support them describe their relationship?
(2) From the perspective of both the individuals with ASD and the supporting individuals, how do their relationships provide support for the individual with ASD?
(3) How does the mode of communication influence the supportive relationship? How do negotiations take place? How are conflicts resolved? In what ways, if at all, are the relationships intimate, reciprocal, and/or mutual?

## 2. Materials and Methods

### 2.1. Participants

Our choices for the types of participants for this study were based on the individuals who originally inspired us to conduct this study. We have attended numerous disability, autism, and education conferences where we have seen individuals with ASD present about themselves, their challenges, and their strengths. Most notably, we found their discussions of supportive relationships very intriguing. We found ourselves wanting to know more about these relationships in their lives and how these relationships provided them support. We felt that much could be learned from exploring the supportive relationships in the lives of successful individuals with ASD. As noted, Bogdan and Taylor [22] suggested that research in special education should focus on “optimistic research”, research that identifies and studies positive examples. We struggled at first with deciding what indicated a “successful” individual with autism. Bogdan and Taylor defined successful as “moving in the right direction and struggling with the right issues” [22] (p. 188). As this definition seemed too vague, we defined success in terms of academic success. For the purposes of this study, academic success means that participants with ASD have been accepted into or have experience in post secondary education, including college, community college, or technical school. The demands of higher education are intense, especially for individuals with challenges in social development, communication, and behavior. Therefore, it was assumed that these individuals had found successful ways of being supported and struggled with challenging aspects of support. Through exploring the experiences of these individuals who have achieved academically, we hoped to understand the aspects of successful supportive relationships for individuals with ASD.

Purposive sampling was used to sample specific individuals who met the criteria of the study. The criteria for individuals with ASD to participate in this study were as follows: (1) a diagnosis of autism by a medical or educational agency not connected to the researcher according to the DSM (III, IV, IV-TR, or 5) or state and/or federal guidelines under the Individuals with Disabilities Education Act; and (2) entrance into and experience in post secondary education, either at a university, community college, or technical school. We did not require that participants provide documentation to show that they met the inclusion criteria of the study; instead we simply asked them if they met the criteria. All of the participants with ASD have presented at conferences about their experiences with ASD and post-secondary education. This helped us confirm that they met the criteria of the study. Criteria for other participants will be discussed later.

Additionally, theoretical sampling was used throughout the study to focus on other participants and experiences that increased the depth of focus of the study [23,24]. Sampling remained flexible throughout the study to ensure “sampling on the basis of the evolving theoretical relevance of concepts” [24] (p. 179). For example, our first two participants with ASD both used an augmentative and alternative form of communication (AAC) and were both female. In order to expand the variation and depth of focus of the study, we sought individuals who spoke as their primary form of communication, as well as male participants.

We began looking for participants with ASD in the same arena where we were first inspired to conduct this study–professional conferences. Although presenting at conferences was not a criterion for participation in this study, we did specifically seek people with ASD who were articulate about their experiences. Also, we sought participants who had developed a conventional way to communicate, either through speech, typing, or writing. We directly approached individuals with ASD at professional conferences to participate in this study.

The participants with ASD in this study used varied methods of communication. Three participants communicated through AAC. One participant was able to type as long as a facilitator was touching his elbow or shoulder. This participant was also able to read his typing out loud while he typed and read the message back after he typed. Another participant was considered an independent typist and did not require any physical touch but did require a supporter to hold the typing device while she typed. The third person required hand-over-hand support to type. We did not use any specific tests to validate this individual’s typing. Instead, we relied on her acceptance in post-secondary education as validation. As well, we documented instances throughout data collection where she clearly showed authorship of her own typing. For example, while typing with a facilitator who did not know our story, she recalled for us how we first met. Finally, the last two participants did not use any augmentative devices for communication and used speech as their primary communication means.

#### Participant Descriptions

Participants were selected based on their willingness and availability to participate in the study. Four of the five participants with ASD were first approached about the study at professional conferences. The remaining participant with ASD was referred to us by a professional colleague. We also contacted each potential participant via phone, e-mail, letter, or face-to-face. When an individual showed interest in participating, we presented him or her with a letter describing the study and detailing what participation involved. Once a participant had agreed to participate, we presented a consent form that included possible risks and benefits of the study. We anticipated that some individuals would be conserved. Therefore, we planned on also seeking the consent of the legal guardian or conservator. However, only one participant was conserved, and for this participant we sought consent from the legal guardian and also had a person witness the typed and verbal assent from the individual with autism. Although we had planned on using pseudonyms, each participant with ASD requested that we use their real name. For them, this was another form of advocacy. Potentially this caused a problem because we were not sure if their supporters would agree to this. They all agreed to this; however, we will only be using the first names of the supporters.

During our first interview with the participants with ASD, we asked them to identify two to four significant individuals who provided them with support. In this respect, participants with ASD served as “key informants” for the selection of the other participants. We anticipated that support people would include parents, teachers, relatives, friends, professionals, and paid staff. The only criterion for selection was that the person with ASD had known the individual for more than 6 months. Once the supporters were identified, we contacted them via phone, mail, or e-mail to ask them to participate in the study. All identified supporters agreed to participate. Overall, there were 22 participants in this study: 5 individuals with autism and 17 individuals identified as significant supports. Of these 17 participants, 15 were female and 2 were male (a father and stepfather). Seven participants were relatives, all parents or stepparents. The other 10 participants were all at one time or another paid support staff. The ages of the supporters ranged from early 20s to mid-60s, and all but two of the supporters were Caucasian. Table 1 includes the name, description, race, and age of each participant at the time of data collection.

### 2.2. Data Collection Methods

Data collection and analysis methods were approved by the Institutional Review Board at the University of San Diego. The primary sources of data in this study included in-depth interviewing and participant observations. Additional sources included documents and other materials such as: published articles or chapters written by the participant with ASD documentaries or other video recordings, conference presentation handouts and/or transcripts, schoolwork, and other miscellaneous documents written by or about the participant with ASD.

#### 2.2.1. Interviews

As we were exploring personal relationships, interviewing allowed us to seek each person’s unique perspective and experience of that relationship. A semi-structured interview guide was used in all initial interviews. Two different interview guides were initially created, one for participants with ASD and one for participants identified as supporters (Appendix A). As the interviews progressed, these guides evolved and expanded according to concepts that emerged from earlier interviews.

Most interviews were face-to-face. One participant with ASD and four support participants were interviewed over the phone due to physical distance, and another support participant requested a questionnaire paired with e-mail correspondence. The number and the duration of interviews ranged widely. Most participants with ASD were interviewed at least two times and a few support participants were interviewed more than once. The total number of interview hours was approximately 60 h. Observational fieldnotes were also written up after each interview. All interviews with participants with ASD were both video and audio recorded. Additional memos were written when these tapes were viewed at a later date. We transcribed all interviews verbatim shortly after each interview. For participants who used AAC as their primary form of communication, detailed fieldnotes were also taken during the interview. The typing that resulted through AAC, which was dictated by the participant with autism, the facilitator, or voice output from a Lightwriter keyboard, was checked for accuracy by reading the sentences back to the participants, as well as listening and watching recorded interview sessions.

#### 2.2.2. Participant Observation

We were able to observe 6 of the 17 dyads studied in person. These observations ranged from 1 to 4 h. These interactions were all video recorded. We were able to observe 4 other dyads through pre-recorded videos or documentaries. Fieldnotes were written up after each observation. An additional source of data was participation and reflection on the developing relationships between the participants and myself. These observations and reflections were captured in memos. This was a very rich source for data and allowed us to experience what we were studying first hand.

#### 2.2.3. Documents and Other Materials

Additionally, documents were collected from participants and used as data. These documents included: published articles or chapters, documentaries or other video recordings, conference presentation handouts and/or transcripts, schoolwork, and other miscellaneous documents. Memos were written up about each document. As well, memos were taken while viewing video recordings. Documentaries and other recordings were also transcribed in order to code. We also kept researcher journals throughout the data collection and analysis process that included analytical, methodological, and personal notes. Table 2 details how each participant participated in the study.

As anticipated, interviewing individuals with ASD posed challenges. Because our participants were “academically successful”, they were very articulate about their experiences and posed fewer difficulties than expected. Interview guides were distributed to participants with ASD via email prior to our meetings. This enabled the participant to become familiar with the questions, provide time to think about how they might respond, and/or prepare responses in advance. If the participant had trouble answering or understanding a question, questions were simplified, restated, and rephrased. Some participants requested that we speak slowly and use as few words as possible. Open-ended questions were used to avoid leading participants toward an answer. However, one participant with ASD had trouble responding to open-ended questions. Therefore, for this participant, we conducted a more structured interview to encourage responses by incorporating more yes/no questions and providing possible response choices. In some cases, vignettes were used to probe for responses.

We remained flexible throughout the study to ensure that participants could respond in the format they preferred. In addition to face-to-face interviews, all participants with ASD corresponded with me via phone or e-mail, with these correspondences being used as data. It was important that we remained available to the participants with ASD throughout the data collection process. We also encouraged participants with ASD to be open and honest. We emphasized that we were not seeking a particular response; instead, we wished to understand their experience and perspective. At the beginning of each interview, we reminded participants that their participation was voluntary and they did not have to answer questions that made them feel uncomfortable.

The interview environment was critical for the participant with ASD; it was important that they felt comfortable and relaxed. We told each participant that we were willing to develop specific and individual accommodations to insure their ease and comfort. Most participants with ASD required breaks during interview sessions. Each decided upon the locations of the interviews. Without exception, participants with ASD requested to be interviewed at home. Interviews with participants who were identified as supporters were conducted in a similar manner, although these participants required fewer accommodations. These interviews took place in the participant’s home or in coffee shops.

### 2.3. Data Analysis

Constructivist grounded theory outlined by Charmaz [25,26] was used to analyze the data. As discussed previously, there were multiple sources of data in this study. We will present data analysis methods used for (1) interviews; (2) observations; and (3) documents and other materials.

#### 2.3.1. Transcripts, Fieldnotes, and Memos

Directly after each interview, we wrote fieldnotes about the interview and the observation that was conducted during the interview. These fieldnotes were developed into memos, which included any descriptive, analytical, methodological, or personal notes regarding the interview and observation. These memos were typed with wide margins so that we could go back and make notes in the margins. All interviews were transcribed verbatim as soon after the interview as possible. Electronic and paper copies were made for all transcripts. All lines were numbered, double spaced, with wide margins for multiple codes.

Memos were written up about each document, video recording, or other material that was provided by my participants. For example, if an individual gave us an article, chapter, or any written material, we wrote a memo for each of them. If an individual provided us a video recording or documentary, we transcribed the recording in order to code the data similarly to interviews, in addition to writing memos.

Data was analyzed throughout the data collection process using the constant comparative method [23]. By coding the data as it was collected, ideas are built inductively and lead the data collection in unforeseen directions [25]. Shortly after the interview was transcribed it was coded. We did not wait until all interviews were done to begin the data analysis process; data analysis occurred concurrently with data collection. Data were coded in two steps. First, initial or opening coding consisted of line-by-line coding. We also coded my fieldnotes and memos, although we did not code line-by-line. Instead, we coded larger chunks of fieldnotes and memos.

After this initial coding session, we wrote a memo that described any analytical, methodological, or personal notes that emerged from the codes. These also contained our thoughts about emerging ideas and patterns. Writing memos allowed us to go beyond simply describing, they allowed us to define patterns.

At a later date, we went back and coded all transcripts a second time. This second step of coding was selective or focused coding where we applied broader codes to larger pieces of data. This type of coding was more conceptual, less open-ended, and a direct result of memo writing. At this time, broader codes were compared using the constant comparative method of grounded theory. Codes were put into categories through comparison of similarities and differences. We found that we had so many different types of participants that comparison and analysis had to take place within stages. We created an analysis plan to guide us during this process. Table 3 details this plan.

The first stage of analysis consisted of analysis within groups. Each group consisted of the individual with ASD and the people they identified as supportive. For example, Sue Rubin, Rita, Aishling, Lisanne and Emily were one group. Within this first stage there were sub-stages of analysis. This consisted of analyzing data by participant type. For example, Sue’s data were analyzed separately as the individual with ASD, Rita’s data were analyzed as a family supporter, and Aishling’s, Lisanne’s, and Emily’s data were analyzed as non-family supporters. When these groups consisted of more than one person, as was the case in Sue’s non-family supporters, this group’s data were also compared. The next step involved comparison and analysis as a group, meaning that the data provided by Sue, Rita, Aishling, Lisanne, and Emily were compared and analyzed together. This stage was repeated for each group. The second stage consisted of analysis among the data provided by each participant category, meaning that all data provided by individuals with ASD were compared, all data provided by family supporters were compared, and all data provided by non-family member supporters were compared. Finally, in stage three the data provided from all these groups were compared and analyzed together.

After stage three, additional memos that focused on broader categories and codes were developed. In these memos, the core categories of the study emerged. These core categories best captured the data and were the beginning steps in creating a substantive grounded theory.

#### 2.3.2. Member Checking

Throughout the data collection process, we checked back with many participants to fill in gaps and further discuss emerging concepts and theories. Member checks ensured that the participants continued to play a role in the analysis of data. Once we began writing the first draft of the results, we again spoke with two participants with ASD and five support participants to discuss the developing grounded theory. This also helped us revise and expand our initial findings. As described earlier in this section, we also sought participants who provided variation among participants. In addition, because data collection and analysis were conducted simultaneously, we were able to seek more information and refine developing ideas in later interviews with many participants.

#### 2.3.3. Integrative Diagramming

Diagramming helped us visualize supportive relationships as a process. This was an invaluable step in data analysis and allowed us to work with larger chunks of data. Through this process, we were able to graphically document my analysis. Diagrams also helped us “gain analytical distance” from the data so that we could see the process more conceptually [28].

#### 2.3.4. Trustworthiness

Trustworthiness, also known as research validity, is critical for confidence in both the methodology and the findings of qualitative research. Goetz and LeCompte [29] defined this term as being concerned with the accuracy of findings. In this study, the researchers followed Wolcott’s [30] recommended nine points to strengthen trustworthiness. Table 4 shows, in the left-hand column each of the nine recommendations. In the right-hand column are the actions of the researchers to promote this dimension of trustworthiness. In addition, two professionals who were considered experts in the field of ASD (have worked in the field of ASD for over 15 years) separately reviewed the codes and verified the findings.

## 3. Results

The data presented in this study are part of a larger data set [31]. The perspective of participants with ASD and the properties they identified as essential aspects of their supportive relationships have been reported in greater detail in Robledo and Donnellan [32]. Herein we focus on *both* the individual with ASD and the individual whom they identified as supportive. These findings of this study combined with the previous report in Robledo and Donnellan [32] complete a full picture of these rich and dynamic relationships.

The following sections will present the core categories of this study: trust, unity, and support. The final section will present the substantive grounded theory that captures the process of the supportive relationships explored in this study. Table 5 provides a summary table of the findings of this study.

### 3.1. Trust

Trust emerged as an essential property of supportive relationships for participants with ASD and their identified supporters. This was a major theme with all participants with ASD. Matthew, who found it very difficult to talk about relationships in his life, spoke about the one thing that he needed in a supportive relationship was to know that he could trust the person who was supporting him. Participants described the need to establish trust within the dyad in order to develop a sense of unity with their supporters. As Peyton described, “Unity is the gin, trust is the tonic”. Only with this trusting relationship as a foundation did individuals with autism and their supporters feel they could give and receive support successfully. If trust was not developed or if it was violated in any way, unity within the relationship was either not developed or was strained. Thus support was greatly affected.

Peyton spent considerable time discussing the importance of trust within supportive relationships during our interviews. Peyton explained that trust cannot be developed or maintained if either person in the relationship does not care for the “advances or growth” of the other person. Additionally, she felt that when the relationship or “union” is in the best interest of the person in need of support (which could be either member), each act that supports that individual to grow makes the trust between the two people stronger. According to Peyton, trust is either “established or shattered” according to how “unified” the two members are in response to situations where support is required for either person. If either member of the relationship is viewed or treated as “gullible” or as a “non-thinker”, trust cannot be developed or maintained. Peyton explained that she has had 21 support staff throughout her life with whom she was not able to establish a trusting and unified relationship. She said that sometimes she knows right away that she will not be able to establish a trusting relationship, while at other times it may take months to know for certain if she will be able to trust a person. Peyton also indicated that she has only been able to establish a “mutually trusting relationship” with four support staff throughout her whole life. For Peyton, trust is not something that people have to prove through actions, rather it is something she “knows and feels” in her heart. She described being able to establish trusting relationships with people by knowing that they are “caring” and always have her “best interests in mind”. Peyton concluded by saying that supporting her along her “journey” is impossible without trust.

#### 3.1.1. Developing Trust

Trust usually develops naturally over time. For some relationships in this study this was the case. However, for many of these relationships, especially in relationships involving paid support staff, the development of trust was established in a different manner. Since the need for constant support was so strong, many times participants with autism found that they did not have time to develop trust slowly with their supporters. Instead, they found they had to force or speed up the development of trust so that support could be successful for them as soon as possible. This was particularly the case when there were changes in staff.

The sense of urgency to develop trust did not diminish the importance of establishing trust, nor make the process any easier. Participants with ASD emphasized that trust within a supportive relationship involved much more than it did in a typical relationship. They knew they were the vulnerable member of the relationship and had more at stake than the other person did. Sue emphasized that trusting someone was vital, yet also involved risk: “Trust is absolutely very important because the really awesome people around me facilitate my life. They are the ones that are responsible for my daily assistance. I’m taking a chance that they are responsible enough to actually run my life”.

#### 3.1.2. Testing for Trust

Trust was not something participants with ASD gave away easily or freely. Trusting someone to be responsible for their lives required a huge leap of faith, and if that trust was ever violated it would take considerable time to re-establish that trust. Therefore, many participants developed strategies to “test for trust”, so that the process could be moved along faster while still ensuring that trust could be established.

Several, but not all participants had strategies to test for trust, yet all participants felt trust was the foundation for building unified supportive relationships. In order to build a relationship, Sue must know that she can trust the person who is going to support her. In order to know this, she puts them through a test. Sue described this test as her way to know how that person will react to her when they are pushed to their limits. Sue stated, “I can’t trust them until I know how they will treat me when they are mad”. Therefore, Sue devised ways to push her staff to their breaking points. These tests were usually specific to whatever upset or bothered the staff person the most. The tests themselves were very intense. Sue can be absolutely ruthless when she is testing a supporter, especially when she is going through an emotional experience, such as when she feels fearful about the transition of staff.

For Aishling, Lisanne, and Emily, Sue’s test involved deliberate physical behaviors displayed in public places, such as head banging, screaming, or throwing her body on the ground, as well as verbal or typed attacks such as, “You’re never going to cut it”. For Sue, this process was an attempt to make her staff very upset, and while they were upset, test them to see how they could support her. Do they still have her best interests in mind? Do they get overly frustrated? All these questions led to one major question for Sue: When you are at your most frustrated state—how will you react to me and will you be able to support me in the manner that I require? In order for Sue to trust supporters, they have to prove to her that they will be there for her when the going gets tough.

Aishling, Lisanne, and Emily all passed these tests with flying colors. Their reaction to Sue’s need to test them for trust showed compassion and understanding. As Emily stated, “That was a really hard time for us to work through, but I knew it was nothing personal. It wasn’t something about me that she didn’t like. I think it had a lot to do with her emotions over transitioning to a new staff person”. They also realized the risk Sue was taking in trusting them, and that she had to develop that trust before she could build a relationship with them. They also knew that this was a way for them to gain Sue’s respect, something they also identified as essential in order for them to develop a relationship with her. Nonetheless, understanding the need for the process did not make it easier. For Emily, it was a very stressful and long process. Her fear was that she would not gain Sue’s trust and would not be able to support her:

It wasn’t that I was hurt because of what she was saying or the names she was calling me. It was pure frustration. Am I going to be able to type with her? Is she ever going to be able to transition and be able to trust me? Are we going to be able to work together? I can’t fail her.

Fortunately, Aishling, Lisanne, and Emily were all able to develop that trust with Sue and this led to the development of very close relationships.

Tyler’s need to test his staff developed later in life. For many years, Tyler was able to develop trust with supporters in a more natural and gradual way. As he said, “It takes time and energy. I need to see that person’s heart. I want to know if they are a loving soul”. However, this past year Tyler’s trust was violated when a staff person sexually abused him. The abuse occurred right after Tyler moved into supported living. Janna stated, “The abuse issue was really huge. It really sucked the life out of Tyler’s trust”. A few months later, Tyler and Janna worked hard at training new school staff for his first semester in college. Two days before classes were to begin one person left without warning. This event only further deflated Tyler’s trust in his support staff. As Janna recalled, “That person had no idea the damage they did in that single act”. His life was like a house of cards. Because of these violations in trust, Tyler felt that he had to test his future staff. His method, in his own words, was to “purposely withhold typing with them to see if they have the interest to deserve my trust”. By withholding typing, Tyler was not communicating with his staff. At the time of our last interview, no new staff members had proven worthy of Tyler’s trust.

Tyler’s method of testing for trust was having a direct effect on his life and his relationships with his staff. His life is full of commitments that require him to type: going to college, presentations, meetings, and advocacy. Because he still wanted to maintain those commitments, he sought out the constant support of his dear friend Janna. As Tyler stated, “I know Janna will do anything for me so I trust her”. Janna, who was completely compassionate and understanding of Tyler’s situation, was driving an hour each way to see Tyler four or five times a week, on top of training his school and supported living staff. This was a schedule that was impossible for her to maintain: “I’ve kept up this pace for three or four months but I can’t do this for the long haul. It’s hard on my children–sleep wise I’m exhausted. And then I feel so bad for him not having a voice so I can never say no to him”. Janna was working with Tyler trying to convince him that his method of testing people had negative implications for his life. She was working with him on developing an alternative method for testing for trust. Tyler realized the importance of what Janna was saying, “I’m willing, but fear is blocking my success right now”. Janna also realized that Tyler was at an emotionally fragile time in his life, and he feared losing her if he began to type with other staff. As Tyler stated, “I’m recovering well, but I still need Janna close”. This process will continue to require support and understanding.

The importance of trust for these individuals was monumental. Although at times they pushed their supporters to the breaking point and required them to meet extraordinary standards, all of the people these individuals identified as significant supporters completely understood and were compassionate about the need to test them. As Lisanne, Sue’s friend stated, “I think with Sue you have to have that level of trust. She had to feel that she was safe with you, and she would test the waters first. She has to trust you”. They understood how much risk was involved and realized that trust must be strong in order to develop and maintain relationships, which served as the foundation of their support.

### 3.2. Unity

Unity was identified as the most essential property of supportive relationships from both participants with ASD and their supporters. In fact, feeling a sense of unity was so critical that many argued that it was not possible for them to give or receive support successfully if that unity did not exist. Peyton described unity occurring between both members of the dyad when each person is “reasserting each other’s values in harmony”. Peyton further defined unity as a “deep connection” that involves intimacy, common interest, and action. Other participants agreed that having mutual, unified, and close relationships with their supporters was the most critical piece to their support. When I asked Stephen how important having a close relationship with the person who supported him was, he stated:

Very important-I think it is key to good support. They need to get to know the individual and know that it is a relationship. That relationship needs to be productive and comfortable. They do need to be qualified and know what they are doing–but the relationship is even more important. Being comfortable with the support you are receiving is important. I don’t care about the politics. If that relationship is not comfortable and productive, in some cases you might be hurting instead of helping.

For Stephen, the quality, level of comfort, and ease within the relationship were all important aspects to a supportive relationship. Tyler described how relationships built on “unconditional love and respect” grounded him so that he could be supported successfully, especially in terms of his communication: “I need that foundation so I can focus on my communication”. Sue described having close relationships with her staff as “unbelievably important” to her support.

Supporters, including staff, friends, and parents, agreed that the connection found in their relationship was the foundation of effective support. Aishling talked about how her relationship with Sue and the support she provided her were inseparable, “You can’t work with Sue without building a relationship first. She won’t respect you. It just won’t work. You have to have the relationship first”. Because of the intimacy that was involved in supporting individuals with autism, Janna did not understand how you could support someone without a mutual connection: “I think it’s a relationship and you get so involved and it’s so personal”. Deborah felt that learning and support could not be separated from the relationship; she felt that she could only support and teach Stephen through her relationship with him.

The relationships that the participants with ASD identified as being significant supports in their lives provided us an amazing group of relationships to explore. The richness, depth, and complexity of these connections were phenomenal. For descriptive purpose, we divided them into two distinct groups. The first group includes staff and friends as support providers, or any individual who is not a family member. The second group consists of family members, which in this study were parents. Because these relationships were so different and at times more complex from non-family member relationships, we felt that they were best described separately. We will briefly describe each relationship knowing full well that we will not be able to capture their full complexity. However, these relationships will be further expanded upon throughout the results section.

#### 3.2.1. Staff and Friend Supporters

This group of individuals consisted of paid support staff (past and present), friends, and colleagues. People did not clearly fall into one category. In fact, most supporters have worn many hats throughout the relationship. For example, Deborah first met Stephen as a personal friend, later became a friend of the family, and eventually became a paid support person as his educational consultant. As a result, these relationships have many facets and dimensions. Of the 10 non-family supporters whom individuals with ASD identified as supports, seven began as paid support staff while the other three started out as friends. However, all non-family supporters at one time or another, even if it was just for a few days, have been paid as support staff.

Those participants who began as paid support staff described the development of their connection in a variety of ways. Five of the seven supporters described building a relationship as a slow, gradual, and often difficult process. Both Emily and Lisanne described developing a relationship with Sue as a gradual process. Emily described that getting used to Sue was more difficult than she expected:

I had a hard time–it’s a hard thing to do–to look at her and separate the behaviors even though I know what was going on mentally for her. How do I treat her? How do I talk to her and help her to do what she wants to do and not talk down to her? There were so many different dynamics going on.

As noted, Sue tested both Lisanne and Emily to determine if she could trust them. This intense and grueling experience provided some obstacles in the development of a friendship. For example, Lisanne described how Sue tested her:

When I first started working with her we couldn’t type sentences. We were trying to type out words. I would say, ‘ok type out bat.’ You know something ridiculous, far below what she does. And then she typed, ‘you’re never going to cut it.’ Totally attacking. She was just trying to see what I could tolerate.

Emily described supporting Sue during this time as a “job”. This was something she felt she had to do so that she would not take things personally. Gradually, over time, Emily began to feel that Sue was beginning to trust her and that their relationship was moving towards friendship, something they both talked about wanting. They began to talk about more personal topics during working hours and started incorporating fun activities into the day such as renting a movie and watching it together. As Emily said, “It was more of us just hanging out and not just focusing on tasks we had to get done that day. Instead we’d make time to just go do fun things together. Just hang out like friends”.

There was one moment when Emily realized that she had established a real connection with Sue and that working with her was no longer just a job. After Emily had worked with Sue for a few months, Sue was faced with a very emotional situation when both of her grandmothers passed away. Emily asked Sue if she could come to the funerals. Sue told her that it was not necessary as the funerals were both on her days off. Emily told Sue that she did not want to come as staff; she wanted to come as her friend. Although this moment meant a lot to Emily, she did not realize what a turning point it was in their relationship until Sue mentioned it in an interview. Emily stated:

I think that was a big thing for Sue–to see that I came on a day off and that it wasn’t just a job to me. In her mind she knew that I came to support her. That really solidified that we had moved past that hard time. I knew it was a big moment for me when I was like ok I’ve invested a lot more into this than I would a normal job. I made a conscious choice at that moment. I realized that it was more than a job. I don’t think I realized how big it was for her until she mentioned that in the interview.

Emily and Sue’s friendship has grown much stronger since then. Sue described Emily as “a good friend who has learned to understand me”. Watching them together gives you the feeling that they truly are close and connected friends. During our interviews they often laughed with each other and bonked heads, which is how they often show affection towards each other.

Lisanne also struggled at first to develop a connection with Sue, but once she did she knew that she had a friend for life: “I can’t ever see my life without her. Once you get into her world and give yourself to her you are stuck. You don’t want to leave”. Sue called Lisanne one of her best friends and still enjoys their time together.

Abby and Sarah both began as paid support staff for Matthew. They found it difficult at first to develop a connection with Matthew, but because they realized its importance for support, they both worked very hard at establishing a friendship with him. Abby described how it took time to develop rapport with Matthew:

At first I didn’t even think he cared that I was there or cared that I would be coming each week. I slowly started to take over the meetings from my supervisor. He started to open up pretty well after that. It was really important to me that he knew I was here to help him with what he wanted help in.

Sarah talked about doing activities with Matthew that he enjoyed so they could develop a relationship, “Matthew and I would get together for social activities, such as going to lunch, going to the zoo, things Matthew enjoyed doing, which hopefully made it more comfortable for him to be around me”. It was very important to Sarah that they develop trust and a level of comfort between them; both of which Matthew mentioned as being essential to his support. Matthew explained, “When others are more at ease with me, I can tell. This makes me more comfortable and relaxed with them”. Abby also described working on developing a relationship with Matthew by letting him know that she was not another authority figure in his life, rather she was someone who was there to support him whenever he needed help. As Abby said, “I just tried to have patience. I just tried to show him that I wasn’t here to stress him out even more”. She accomplished this by having a very relaxed and calming manner. After working with Matthew for over three years, Abby developed a close bond with Matthew. As she stated, “Matthew will always have a special place in my heart”.

Claire, who supported Stephen in high school, explained how their relationship started to develop once they started doing activities together outside of school. For example, “He told me he had never been on a rollercoaster before. So we went down to Mission Beach and rode the rollercoaster”. Because Stephen had a lot of trouble with social skills, Claire credits the development of their relationship to her taking an interest in Stephen’s interests. This allowed them to connect at the friendship level. As Claire stated:

I think more than anything he liked the fact that I became involved in his life. He didn’t have friends in high school and his brother’s friends were just too involved in their own thing to involve him. So I think I was somebody who took an interest with him and was willing to do things with him outside of school.

Although Claire no longer works with Stephen, she continues to remain in Stephen’s life as a friend, and they often get together for coffee to talk. Claire described their current relationship: “We are both busy with life and we see each other when we can, like normal friends”.

Two of the seven supporters who started as paid support staff described developing a relationship with the individuals they work with as an easy and enjoyable experience. Mary, who supported Peyton, described feeling an immediate bond from the start. Mary stated, “I was so impressed and intrigued by her and she was willing to let me into her life. It was an instant bond”. Janna, had a similar experience with Tyler, “I fell in love with him in about 10 seconds”. Tyler also described their connection as “instantaneous”.

Of all the non-family member relationships, that between Janna and Tyler stood out as being exceptionally close and connected. Janna started working with Tyler as his facilitated communication facilitator and trainer when Tyler was in middle school. In Tyler’s words, “I’ve had my angel since 6th grade”. Within a short time, Janna realized how committed she had become to Tyler: “I was with him for life! I just really fell in love with him and I really cared about Lynn and just felt there was no way I was walking away from that situation–there just wasn’t a chance in the world. So I’ve been committed for life basically”. Janna and Tyler could not have said nicer things about one another. They both described many aspects of their relationships that make it unique, including two very important aspects– humor and spirituality. Tyler and Janna share a similar sense of humor, one that is full of sarcasm. As Janna stated, “We can get going on these one-liners and never stop. People have told us that we should be a comedy act!” Another important aspect to their connection is spirituality. This is something that they both felt was an essential element. Tyler stated, “I think Janna and I share common spiritual views and that also makes us extra close. We know there is a higher power with a plan for us together. We let that guide our work together”. The connection that Janna and Tyler have with each other has only become stronger over the years and has extended to both of their families. They both feel that in many ways they were destined to be together. Janna beautifully captured how significant her relationship to Tyler is: “You know I think in your life when you end up on your deathbed you could probably count on one hand your true and real friends, and he really is one of my true and dearest friends”. Tyler also captured how much the relationship he has with Janna means to him: “Her support feels like your favorite blanket that you snuggle at night. Never leave home without it”.

Three of the 10 supporters first developed friendships with the individual with autism and later moved into more formal paid support positions. As noted, Deborah developed a friendship with Stephen that developed into family connections and eventually a paid support position as his educational consultant. Deborah described Stephen as “a friend unlike any other friend”. Over time their relationship became more and more comfortable. Deborah explained, “He would always come over for parties and he would just be here. He would just come over and talk and come for dinner”. Deborah felt that Stephen knew he could come to her or her family with anything: “I feel as though he has developed that sense that if there is a problem he knows he can come here–he knows that we are a safety net for him and we always will be”. Stephen described Deborah as someone who has been a great support to him: “She is my educational consultant and also a personal friend of mine. She does a lot of things for me. Oddly enough, you know what they say that God sometimes puts you with the right people. And that was true with Deborah”.

Martha first developed a relationship with Peyton based on their similar interests. Martha recalled, “I got to know her because her interests in life were of interest to me. I saw her as a person who had the potential to communicate more effectively and her experiences in life interested me. That was really the basis of our relationship”. Martha explained how this has helped maintain the friendship, “I respect her experiences and knowledge and I think she respects mine and we like each other”. Martha felt that mutual respect and affection maintained their relationship. Last year, Peyton added another dimension to their relationship when she asked Martha to be a paid support for her for a 5-day period. Except for that short period of paid support, Martha primarily has shared a friendship with Peyton.

Aishling developed a friendship with Sue prior to becoming a paid staff person and now has gone back to the role as primarily her friend. Aishling first met Sue when they were both students in high school. Aishling described:

I heard about Sue before I met Sue. I heard that we had this individual with autism coming into our class. We would hear Sue screaming in the hall and we watched a video about her. About a month, maybe two months later she came in for about 30 seconds, screamed and ran out. She kept coming back like that until she was able to sit in the chair and relax. I asked if I could join her group because we were doing these projects and people were starting to walk away from her, and I thought that sucks, so I walked up and asked her if I could join her group. She said sure and that was basically the beginning of the end!

The connection between Aishling and Sue grew into a very deep and loving friendship. In 2004, their relationship was documented in the academy award nominated CNN documentary that Sue wrote about her life called *Autism is a World* [33]. In the documentary, Sue described their relationship: “Aishling and I have a dear friendship that has spanned 12 great years and many more to come. She is a true friend and both loves and antagonizes me like the sister I never had”. Aishling viewed their relationship similarly; she sees Sue as a sister, “There’s this part of your heart that you share together. It’s more like a sisterly relationship. Lisanne, Sue, and I really are a sisterhood. I mean we were a family. We were a tight unit–we had each other’s backs and that was the way it was”.

Aishling worked with Sue for over 7 years as her school support. She never viewed working with Sue as a job; it was always a relationship. When Aishling left the job to pursue a career as a special educator, it was devastating for Sue. The beginning of this transition was documented in Sue’s documentary *Autism is a World.* Although Aishling admitted that this time was very difficult on both of them, Aishling felt that the documentary left viewers with the sense that she was going to disappear from Sue’s life. The opposite was true. Aishling described her relationship with Sue as being closer now that she no longer works with Sue:

It’s better now because we are friends. And we are strictly friends. No one is working for anyone. It is there because of who she is and what we mean to each other. I think the movie portrayed it like, ok now what happens? Well now you get a life with friends, not a life with friends who get paid. I think it is more open and honest and more raw. It’s so simple and it’s so pure and it’s so untainted by anything. I think our relationship is more important to her now than it was back then. I am here because I want to be, I am a part of her life because I want to be. That’s what it is now. It’s proof. There was never an end. It was a beginning in a way.

#### 3.2.2. Family Supporters

Each participant with ASD identified their mothers as being a significant support in their lives and some indicated that both of their parents played this role. Exploring these relationships was a complex process. Just as in any parent-child relationship, many changes take place over time that affect the nature of the relationship. Again, we will briefly describe these relationships knowing full well that we will not be able to capture their full complexity.

Rita, Sue’s mother, was the first person that Sue identified as a significant support in her life. Sue wrote: “Rita is awesome. She is the reason I am able to fight my autism. Actually, Rita is my mom and my friend also”. Rita also described them as being very close, “Sometimes I say we have two bodies but we only have one mind. We almost have a mind mold because we are so close”.

Rita’s relationship with Sue has undergone many changes. When Sue was an infant, Rita recalled that developing a connection with Sue as hard: “It was difficult not getting eye contact or hearing her laugh. I’m sure she didn’t recognize us as her parents as opposed to someone else”. Regardless of the seeming lack of reciprocity, Rita made Sue a part of the family by always including her in family activities. The relationship between Sue and Rita forever changed when Sue started communicating through facilitated communication. Rita discussed how her ability to communicate changed Sue’s role in the family:

It was interesting to learn who she was as a person because we didn’t know before. That really changed our relationship. So since she could communicate we would have her participate in family discussions, which she couldn’t do before. I think that we loved her as a retarded person, but when she showed us that she was bright there was just so much more that she could offer us even if she wasn’t hugging or kissing us–intellectually she could participate in the family.

Another major change took place within their relationship when Sue moved out of her family’s home. Rita was no longer Sue’s primary support provider. However, both she and Sue felt that this has made the connection between them stronger. Sue explained that their relationship is more like a friendship “because time spent with her is now because we like each other”. Rita described how they continue to be close and how Sue’s independent living has added new dimensions to their relationship:

I know she does things with her friends that I don’t know about and I hear about them later. I think it’s actually good because by the time a person reaches 18 or 19 it’s completely natural that they have secrets from their mother. It kind of gives me a thrill because that’s the way it should be.

Aishling described the beauty she observed in the connection between Sue and Rita: “There is just this love and adoration for each other. There is just this mutual respect for two women who now understand each other. It’s beautiful”. Sue and Rita talk about each other with immense respect and love. For example, Sue wrote: “My mother is my strength. She has devoted her life to my success and to the education of people around the globe about autism. I only wish that someday I can be half the woman she is and pray that every daughter in the world is as blessed as I am” [34] (p. 108).

Matthew identified his mother Nancy and his stepfather Tom as being significant supports in his life. Nancy is always the first person that Matthew goes to when he needs help. Other supporters whom Matthew identified spoke about Nancy being the closest person in the world to Matthew. Although Matthew also identified Tom as a significant support, Tom spoke about how he was always second to Nancy, “Nancy has been the primary parent”. Tom recalled how he was there for Matthew in any way he could be, but Nancy was always the person that Matthew wanted first. He recalled many instances of Matthew coming into the house saying, “Where’s Nancy?” Nancy described her relationship with Matthew as so close that sometimes she has “difficulty stepping back and really seeing who Matthew is as a person”. As an infant, Matthew would not make eye contact with her and did not express any signs of separation anxiety. Regardless, Nancy described her connection with Matthew as “extremely close”. Nancy has always been very proud of Matthew’s intellectual abilities and felt it helped them develop a close bond. Now that Matthew is older and living on his own, Nancy described their relationship as being more on an “adult level”. Matthew no longer lets Nancy “boss him around” and when they spend time together, it is to do fun activities that they both enjoy.

Tyler identified his mother Lynn as a significant support in his life, as his “dearest love” and “partner in crime”. Lynn and Tyler have always had an extremely close bond. As a child, Tyler showed a lot of physical affection towards Lynn, which made it very easy for Lynn to form a close connection with Tyler. While he was in grade school, Lynn described their relationship and her efforts to support him as her “major focus in life”. Over time, she began to worry that possibly they were becoming “too close” and feared that if they remained this close Tyler would become too dependent on her and neither one of them would be able to have an identity outside of the relationship. In order to create more separation and to ease some financial issues, Tyler moved away from Lynn into a group home when he was 16. This time was very hard on both of them. Lynn recalled going through both depression and empty nest syndrome. Over time, both Lynn and Tyler were able to form identifies independent of each other yet still remain very close. They are very active in each other’s lives and advocacy.

Stephen described his Mom, Liz, as playing a major role in his support. She was there whenever he needed support, and she was someone he could “always count on”. Liz described having a deep connection with Stephen since his infancy. Although there were many challenges, Stephen was very interactive and affectionate with his mother. Although Stephen spoke at an early age, he did not usually share words of affection such as, “I love you”. Nevertheless, his actions and behaviors let Liz know that they had a connection: “Even though he wouldn’t say stuff he was always funny and giggled and you knew there was an interaction–a connection”. As the years went on Liz and Stephen developed a typical mother-son relationship. During our interaction he complained about how her “constant need to clean” or her disruption of his schedule upset him, but overall we observed this to be a very typical mother-son relationship. Talking with them together, we could see how similar they were and how they enjoyed similar things, such as having vigorous political debates. When Stephen is in need of emotional support, he always goes to his Mom for love and support. Liz stated, “No matter how much he complains about me getting on him about something, whenever he is upset he comes home and I provide him with the comfort he needs”. Liz felt that their journey through life together has made them extremely close: “I wouldn’t change it for the world. I don’t know how other families feel but Stephen is–I just love him to death. We have this really great relationship”.

Peyton identified her parents Dianne and Pat as being the “most amazing gifts” to her life. They have been Peyton’s major supporters for her entire life. They are very different people and Peyton described having very different relationships with each of them. Dianne described the connection she shares with Peyton as “extremely close” and that being one of her primary supporters has only helped further develop that connection: “Peyton and I can really talk about anything. I know that she talks to me from the heart. I can tell her anything and I know she tells me anything. When you go through tough times it just really brings you close, so close”. Pat and Peyton have a very close father-daughter bond. They have spent a lot of time developing that bond through outdoor activities such as walks and bike rides. Pat described the love that he has for his daughter, “I have a huge love, adoration, and respect for her. Because of who she is and what she’s done, because she’s my daughter. There’s such as affection that I feel fortunate that she’s my daughter”. Although Peyton, Pat, and Dianne spend much of the day together they have been able to each maintain their own unique identities. Pat and Dianne view Peyton as an adult that they are blessed to have a relationship with.

#### 3.2.3. Reciprocal Nature of Relationships

The reciprocity involved in these relationships was an aspect that many participants felt very strongly that we should understand. Both the participants with ASD and the supporters realized that at first glance these relationships did not look reciprocal. Rather, they looked so very one-sided that it appeared the supporter was only giving to the individual with ASD and receiving nothing in return. What we discovered was that many of these participants felt that the individual with ASD gave them back much more than they could return. Yet, what they received was qualitatively different from what they gave. Many of the participants described how the individual with autism gave things back to them that they could never find in another relationship. The following section describes the major properties of reciprocity that occurred within these relationships including: (1) intrigue and uniqueness; (2) friendship; (3) affection and love; (4) sensing emotions; (5) spirituality; (6) influence and advice; (7) learning and growth; (8) providing a focus; (9) inspiration; and (10) pride.

• Intrigue and Uniqueness

Many participants felt that it was a very special gift to have such an intriguing and unique person in their lives and that they really enjoy getting to know them. Liz was also very fascinated and interested in Stephen and was intrigued by his abilities to program a computer at the age of 4 and act like a “little engineer” while he was growing up. Liz recalled that although Stephen may have difficulty with social skills and communication, he has many other interesting skills: “When he walks into a room he’s not going to be able to tell you what people are doing, but he will be able to tell you every thing about their wiring”. Rita also appreciated the uniqueness of her daughter Sue. She struggled to find the words but finally said, “I’d say she’s really an interesting person and it’s just a different experience. The rest of us are essentially alike, I mean everyone’s different but we are essentially the same. When you are with her she’s just different and more interesting than the rest of us”. Aishling and Lisanne also appreciated Sue’s uniqueness. Aishling emphasized, “There’s no one like Sue! I could never have this relationship with anyone but Sue”.

• Friendship

Participants described the significance of the friendships they shared with the people they supported. Martha talked about getting things back from her friendship with Peyton that she could not find in other relationships:

I’d just like to stress that as a friend, although she requires kind of unusual supports–you have to orchestrate the environment, interpret her behaviors, go through all the metaphors–those are kind of unusual supports that may make people think that as a friend that I’m doing all the supporting, but in fact, she is a good person to have as a friend because she is able to provide, in her own way, unusual supports to her friends as well that they don’t usually find in other friendships.

Martha further explained that because Peyton knew that she was interested in Peyton’s life experiences, Peyton would give her details about her experiences that most friends would not, thereby providing Martha with insights that she knew Martha would find interesting.

Janna was quick to let me know that her relationship with Tyler is very reciprocal, “It’s a two-way relationship. As much as I give, I get back, and much more back. So he may think ‘I need her’, but I need him too. He’s a good friend, and I can always count on him for his friendship”. Janna recalled many examples of Tyler’s supporting her when she was in need of a friend. For example, when Janna felt completely humiliated by a colleague, Tyler risked his own relationship with that colleague to stand up for Janna. Janna described how much that moment meant to her, “I will never forget that. That was so nice of Tyler to put himself out there for me”. On the same day, Tyler gave up a social engagement to support Janna when she was upset, “He just knew I needed support. He wanted to spend time with me. What a great friend”.

Claire described how open Stephen was to developing a friendship with her: “He likes you for who you are and doesn’t expect you to change who you are. He is open to friendships with anyone”. Deborah also described how Stephen was enthusiastic about forming a friendship with her:

I first met Stephen when he was working in the sound booth at a performing arts center. Stephen was working the lights and I was working the sound. He was so kind to me and to everyone. He wanted to feel connected. He was more than willing to show me the ropes and help me out in any way he could.

• Affection and Love

Many participants described how reinforcing it was to get signs of love and affection from the people they supported. Lisanne frequently mentioned examples of physical affection that she shared with Sue and how meaningful those interactions were to her:

She sits right next to you and she puts her hand on your lap. And it’s just that–she’s reaching out and making that physical connection. People with autism aren’t supposed to do that! It’s those little things that you get from her and it’s at that moment that it’s all worth it.

Both Aishling and Lisanne described seeing love and affection in Sue’s eyes. Lisanne explained the connection she receives from Sue’s eyes, “Those eyes! You know when you look into those eyes and you know she’s giving back to you. She may not be able to say it or she may not be able to come up and hug you but you know she feels it”. Aishling added, “A look from Sue is worth a thousand words”. Sue also talked about how her eyes are a reliable way to express her emotions and thoughts to friends who know her well:

I am very fortunate that my friends and family are people who know me very intimately. Many times I feel as if oral communication is over rated. Much of how I express myself is through my eyes. Those close to me are easily able to tell if I am sick, tired, or happy, by just looking at my face. My expressions are not always appropriate yet my eyes are the windows to my soul.

[34] (p. 86)

Both Lynn and Janna enjoy Tyler’s physical affection. He even calls himself the “hug monster”. During our interview with Tyler, Tyler leaned over and gave Janna a big hug and kiss when he was describing how much her friendship meant to him. Mary described about how she and Peyton always say hello with kisses and hugs and this lets Mary know that they are “close to each other”.

• Sensing Emotions

Sue, Tyler, and Peyton all mentioned that they could sense the emotions of their supporters. Tyler described it as “seeing people’s energy fields” and being able to tell how their “energy melds” with his. Peyton said she was able to see “auras” around people and “sense their emotional state”. Sue also mentioned that she was able to sense her staff’s emotions. Both Peyton and Sue described this as often being very distracting and could get in the way of receiving support. Therefore, they encourage staff to be very open with them about their emotions so that it can be resolved and they can focus. Tyler reported that sensing the emotions of his staff did not distract him; however, if he sensed that a staff member was upset, he felt compelled to help them.

Supporters talked about the positive and negative aspects of knowing that the person they supported could sense their emotional state. On a positive note, many participants enjoyed having complete honesty with the person they supported, something that many felt they could only have in this relationship. Janna recalled, “I can’t hide anything from him. He’s a good friend. I never try to hide things from him anymore. I just show up and I’m here–all of me–he knows what’s going on so why deny it, just be honest”. During one interview Tyler ended the interview early because he told us that Janna was “running on empty”. This was not something we could have picked up, but Janna confirmed that she was feeling this way. However, sometimes supporters wanted to keep some emotions personal. Lynn understood that she could not hide emotional feelings from Tyler, but that was often hard on her. Sometimes there were emotions that she did not want him to know about, such as stress, discouragement, or depression, which she wanted to keep to herself.

Emily admitted that she used to get “caught up in little details and wallow about things” in her life, but having such honesty in her relationship with Sue has caused her to view things differently. Emily described:

If we bring anything into the house it affects her too, she can feel it. It makes her upset. So I have to consciously not bring it into the house. I think to myself, I can deal with things later. And so I put things in the back of my head and I end up not worrying about it later because I realize it’s really not that big of deal.

Because the individuals they worked with could sense their emotions, supporters had to be completely honest and upfront about their feelings. Supporters described this as primarily a positive aspect of their relationship because they felt they could really be themselves in these relationships and that there was nothing that they should or could hide from the people they supported.

• Spirituality

Supporters described how the people they support have often brought a spiritual element into their lives. Tyler brought up this aspect of his relationship with Janna in our interview without our asking. Tyler talked about how this spiritual connection made them “extra close”. He explained how they talked about miracles in everyday life, communicated without words, and shared some common spiritual gifts. Tyler felt that this spiritual connection they shared helps guide them on their “mission together”. Janna spoke about how this connection was so important to her and was something she could not find with anyone else: “Tyler and I share that common ground so we can really talk about things that I’m interested in and that he’s interested in. It’s really nice to have a place to talk about that. It’s important to him too”.

Both Liz and Lynn spoke of how their relationships with their sons have led them to be more spiritual people. Both feel a strong sense of guidance and purpose from a higher being. Pat and Dianne talked about Peyton’s interests in eastern philosophy and spirituality. Although they felt they have a very limited understanding of it, they have been sure to provide Peyton with other supporters with whom she can talk with about her spirituality. As Pat said, “For us it was whether to embrace that or reject that. But this is her reality and we accept that”.

• Influence and Advice

Participants reported that the people they supported influenced their lives and gave them advice. Sue called this her “specialty” in giving back to her friends: “Loneliness no longer is a part of my life. My support people ask me to spend time with them when they are not working. Mopping up their problems is my specialty. They respect my advice and enjoy being with me” [35] (p. 422). Aishling, Lisanne, and Emily all agreed with this statement and explained how much influence Sue has on their lives. Aishling talked about things in her life not being “real” to her until she shared them with Sue: “Sue has to be there or else things are not real for me. Like my graduation and now my engagement party–it wasn’t real until I was sharing it with Sue”. Emily described how Sue offers her unique perspectives and advice. Emily is very appreciative of Sue’s openness and honesty: “She doesn’t use flowery phrases. She’s filled the void of finding someone who will just tell it like it is”. Emily went on to say how much Sue’s advice means to her:

The stuff she gives to me and helps me with is so much bigger than the daily routine stuff I do for her. I don’t think you can put those things on a balance and say it’s equal, but there is a lot of give and take in the relationship and it’s very important to each of us.

Sue added that she advises her friends by “telling them what they know but don’t want to admit”.

Janna and Lynn also mentioned that they often go to Tyler for advice. Lynn called him her “cheerleader”. She said: “He’s like a motivational coach sometimes. He’ll remind me to stay open for guidance and support from a force that’s beyond us”. Janna also mentioned that Tyler gives “great advice”.

• Learning and Growth

Pat described his daughter Peyton as a “great teacher”. Both Dianne and Pat feel that having Peyton in their lives has given them the opportunity to learn and grow:

Peyton is the greatest thing. I mean it’s a relationship. Certainly there’s love but there’s a completeness and a satisfaction to it. I just can’t think of having a better or more fulfilling relationship. Somehow in the process you learn a lot more that makes it more wondrous and makes you more appreciative rather than seemingly knowledgeable.

Pat and Dianne both feel that they have been able to learn so much from Peyton because they have always been open to growing with her. Pat stated, “We’ve wondered if we would ever have had this kind of growth without Peyton”.

Lynn talked about learning about who she was and finding her own voice through her relationship with Tyler. She so beautifully captured her transformation:

He’s literally transformed me. He’s helped me grow in ways I never thought possible. I mean I attribute him to helping me find my own voice because frankly I think I fit more of the doormat personality before where I was adapting more to what other people would expect. I would adapt more to what other people wanted from me rather than having my own voice. And so clearly this whole struggle that we have faced together has formulated my own voice. So in our journey to find his voice, I found my own. That’s the irony of the whole situation.

In many ways by supporting and having a deep connection with Tyler, Lynn was able to support and know herself better.

Aishling also discussed how she grew through her connection with Sue: If you think you are going to help Sue then you’ve got another thing coming. Sue teaches you who you are. Sue is the person who has helped stabilize me and has helped me realize the person I am. I don’t mean she told you who you were but in the act of knowing Sue that’s when I knew myself better.

In order to go through this transformation, Aishling felt she had to be open to allowing Sue to influence her life. She stated, “If I wasn’t willing to really let Sue in my life. I wonder if I would be the same person I am today. Probably not”.

Claire described how she learned from Stephen’s interests and activities: He’s very knowledgeable on lots of interesting topics. I mean it always fascinates me how involved he is in campus life. He’s involved with issues at school, politics, everything. I always enjoyed those topics of conversation because he made me more involved in those things when I wasn’t. In some ways he was wiser than I was. I really learned a lot from him.

• Providing a Focus

Liz, Nancy, and Lynn detailed how their sons have provided them a focus in life, which has proven to be a very rewarding experience for each of them. Nancy described this dimension of her life as very rewarding: “It’s given me a huge focus in my life. I’m now the Vice-President of the Autism Society Wisconsin Chapter. I also run a social group for people with autism. It’s been a gift to me in that way”. Liz explained how advocacy is a huge passion in her life now, all inspired by Stephen. This was a common theme among all parents. Each had made advocacy and teaching their children how to advocate for themselves and others a huge focus in their lives. Lynn described how her advocacy work has inspired Tyler:

Tyler observed me as an advocate all the way along in different ways and heard me talking or saw me reading–even if I didn’t talk about it directly, it was our lives together. And so now he’s become an incredible self-advocate. I mean the kids do what the model does. We model what our kids pick up and then they carry it on the next road.

• Inspiration

Supporters talked about how inspiring these individuals were and how this affected their lives. Abby described how Matthew has been an inspiration to her: “Matthew really defies what you think about someone who typically has autism. He’s struggling but he’s doing so well. It was really inspirational to see him and to take in all his abilities”. Abby went on to say that she could not imagine her college experience without Matthew. When Abby found herself feelings overwhelmed with the stresses of college, Matthew’s success in college kept her going.

Claire described how knowing and working with Stephen inspired her to become a special educator, “I learned from him that even people with significant challenges can still be successful, and he was the first kid I got to see that happen with. So I knew it could be possible for others”. Deborah also talked about how Stephen was not only an inspiration to her, but to her whole family, “It’s refreshing and inspirational to see someone in life who has so many challenges and stretches himself to work through them all. That motivation to learn is addicting and inspirational”.

Emily discussed how knowing Sue has inspired her to rethink her initial reactions to people who may appear different, “Just knowing her and all that’s going on inside her contrasting that to how she looks just makes me look at all people differently. I would have thought I was a very open minded person before knowing Sue, but now I always take a second look”.

• Pride

In 2005, Sue’s documentary *Autism is a World* [33], which was written by Sue, received an academy award nomination. Rita, Aishling, and Lisanne all described how proud they were of Sue and how they too were enjoying Sue’s “celebrity” status. They were all quick to point out that it is Sue who should have all the praise, and they were just lucky to play a role in her success. Besides walking down the red carpet at the Academy Awards, Rita recalled a memorable moment when someone they did not know approached them on the street to tell them how wonderful the documentary was. “That was kind of great–to be walking out of a restaurant in Beverly Hills and to be recognized and approached. That was really special for both of us”. Rita added, “I am always very proud of Sue when she presents because she always makes an impact wherever she is presenting. She does so well and that’s a reflection on me, it’s a very positive thing”. Aishling described having a similar feeling when she went to conferences with Sue: “The fact that you can be a part of that and watch it happen is very humbling. I’ll compare it to motherhood, like when you hear about mothers and the humility they feel towards their children’s success. I felt like that, like I helped create this. I had a part in this”. Aishling was very honored to have had that opportunity to witness Sue’s growth over the 13 years of their friendship.

Tom also described feeling proud that he had played a role in helping Matthew become “an adult and productive member of society”. Deborah felt prideful that her family provides Stephen with a “safety net” in his life that he can always count on. She realized that so few people have these “safe havens”, and she is glad she has been able to provide that for Stephen.

### 3.3. Support

Properties of successful support emerged during the exploration of these supportive relationships. They primarily emerged from examples and explanations of how support was given and received within these relationships. The properties are also based on positive and negative experiences of support that may have occurred outside of these relationships, yet still impacted the individual’s life. Although negative experiences of support were not the focus of this study, participants with ASD felt that some negative experiences provided them an opportunity to learn more about their own support needs. The following emerged as essential properties within the supportive relationships identified in this study: (1) shared vision of independence; (2) presuming competence; (3) understanding; (4) inclusion; (5) communication; (6) collaboration; (7) consistency and flexibility; and (8) personal characteristics and interaction styles. These elements are described below.

#### 3.3.1. Shared Vision of Independence

The ultimate goal of support for all of the relationships explored in this study was independence. Both participants with autism and the people who supported them agreed that the goal of independence needed to be a “shared vision” between both members of the dyad. This vision served as both the foundation and driving force behind all support. Both participants with ASD and their supporters described independence as a process. Individuals with ASD sensed that in many aspects of their lives they will never be totally independent, yet they want ultimately to be as independent as they can be. As well, they desired to constantly push themselves and be pushed by their supporters towards greater independence. As Sue pointed out, “I want to be as independent as I am able to be”. Participants with autism desired support in their journey towards independence. This process will be further explained in this section by describing the role each type of participant plays in this process.

On the path towards independence, participants with ASD emphasized the vital importance of having control over their own lives and having the final say in all decisions that affected them. Many felt that much of their lives were outside of their control due to the challenges that ASD presented to them. Their voices, behaviors, and movements were aspects of their life they reported having little control over. Therefore, they sought control over any aspect of their lives that they could. Many felt that support and relationships were areas where they could exhibit more control. Pat recalled how Peyton communicated with him about her need for control within her life.

She wanted to be in control of her life—she didn’t want to be in control of other people’s lives but she wanted to be in control of her life. She wanted to be able to say ‘no!’ whether that is to a decision that impacts her life or to someone that is threatening her. You can do something 9 out of 10 times and if that 10th time for some reason it seemed to be more critical to the support person in controlling Peyton—well that 10th time is something that takes the rug out from underneath her.

Peyton, and other participants, talked about the importance of support being person-centered, however, they felt that this meant more than their respective support team merely having their best interests in mind. They themselves needed to make the major choices within their own lives. They wanted to be more than just an equal member on their support team. If there was something in their life that they could have a say in or have control over, than they wanted to ensure they had it.

Many participants felt they did have this role in their relationships that they identified in this study as supportive, and that this has played a major role in their success. Tyler talked about how his Mom has let him make major decisions for himself since grade school. These decisions have ranged from whether or not he should have facial hair to major medical decisions. For example, when Tyler was in high school, doctor’s found a growth near Tyler’s brain. Although the growth was not cancerous and did not necessarily need to be removed, Lynn allowed Tyler to decide if he wanted it removed or not. Lynn described:

If it were my choice I probably would say don’t do the surgery. He said he wanted it out. So that’s what we did. It was his decision. So when he does things I wouldn’t do, those are good indicators that he’s making choices for himself.

Stephen felt he had a lot of control over most of his life, especially when it came to his support: There may be some things in my life I may not want the support team to know about or I don’t need support in everything. There may be some things that I need help with and things like that, but I am selective in who I want to do it or who I want to know about it.

Stephen also said that he has gotten to this point by learning from negative experiences in his life where he felt he was being controlled. For example, Stephen described how he did not have control over the people who worked with him in grade school:

I advocated for myself and got a word processor and some other tools and an aide. Some of which I liked, some of the aides I didn’t like. I’ve advocated that the school system often doesn’t do a very good job of matching an aide with who the person really is. Someone else does the interviewing; someone else does the hiring and the firing. I wish I had more control over that aspect of my life when I was in elementary and junior high school.

Sometimes Stephen felt that his life was being controlled by his support team rather than their being “passengers assisting me to be the driver of my own life path”. Stephen continues to believe this is important not only for him, but for all individuals with disabilities. A major focus of his advocacy work is dedicated to advocating for individuals to be in control of their life choices. As Stephen said, “Others shouldn’t assume what people want to do with their lives”.

Recently, Peyton has had serious health problems. Due to these health issues, she had not been able to perform skills that she had easily been able to do before. Nonetheless, Peyton was adamant that she have control over all of her health decisions. This was a choice that her parents, Pat and Dianne, supported completely. Pat talked about this process, “She’s been responsible for every medical decision and every medication withdrawal issue and she’s determined what she wants to do, and like so many things she has been very brave and very committed”. Even when Peyton is having health problems, and at times may be unable to feed herself, she stills desires to have control of whatever aspects of her life she can. Her parents and other supporters recognize that and respect that desire.

Participants with ASD spoke about how they were best supported when supporters let them first try things independently and step in only when support was required. They talked about how being “over-supported” was something that was extremely frustrating to them and did not assist them towards their goal of independence. When I asked Tyler how his Mom supported him he said that she let him “make mistakes”, which he felt assisted him in his process toward independence. Stephen talked about the importance of support not making him “100% perfect”, because he felt he had learned the most from the times he had failed. As he said, “There is a learning experience involved with getting things wrong. If an aide is so intrusive that the person always get an A because of their assistance–that creates dependence. ” Sue captured how she needs to be supported toward independence:

Living on my own with the help of others has given me far greater independence than my parents or I ever expected. My staff push me to be able to do things with the least amount of support necessary. They are constantly teaching me that I must rely on myself first and then ask for aid if I am not able to accomplish something on my own. I have experienced problems with staff on whom I become co-dependent. I find that I am happier being tested to see what my strengths and weaknesses actually are. I am not afraid at all to ask for help from my staff and friends because they are truly there for the purpose of aiding me in my times of need. I feel much more independent than I could have ever imagined, and that feeling alone is intensely gratifying.

[34] (p. 94)

In order to provide support like this, supporters talked about the constant need to check for competencies and push the people they support towards independence. The rule of thumb seemed to be: assume they can do something on their own until they prove you wrong. Pat talked about how he always assumes Peyton can do something until she shows she needs support: “I err on testing her to where she cannot definitely do what I thought or what I would have hoped she could do or had seen her do. So there’s a kind of retreat in figuring out what she can and can’t do and then you fill in and give her that support”. Pat explained how supporting Peyton while riding a bike served as a metaphor for how he supported Peyton, “I think it’s where she gets the most freedom of anything she does because she knows that she is in control and yet she needs me to touch her shoulder if she starts to hooch out onto the road”.

For parents, supporting and promoting their children’s independence was a complicated process. A similar trajectory emerged from the stories that each parent told. During infancy and early childhood, parents were extremely involved in their children’s support. Through those early years they created strong and loving relationships, which served as a strong support foundation for their children. As time went on, parents realized that they must slowly let go of the control they possessed over their children’s life and begin encouraging and supporting their children’s independence. Some parents had this realization when their children were in grade school, high school, or college. Nonetheless, each parent in this study realized that in order for their child to ever have a shot at adult independence, they had to cease controlling every aspect of their child’s life. Letting go for these parents involved a completely different process than letting go of control in a non-disabled child’s life. As Pat said, “It’s not the kind of situation where you throw the kid in the water and hope they’ll learn to swim”. Liz described this “letting go” as “discovering a balance” where you could still give the required support while at the same time provide the space and means to move towards independence. This process continues for all parents involved in this study. The stories of Nancy and Lynn will illustrate this process.

When Matthew was young, Nancy described herself as being “very involved in his support”. She was determined to find supports and resources for him. She enrolled Matthew in numerous research studies with the hope that this would provide him with the latest therapies. Nancy stated, “That was when Matthew become involved in research studies because I was always a believer that if you were in a research study that you would get resources available that you would not get otherwise. You find out what the current thinking is about autism, which is helpful”. When Matthew was in grade school and he began to work more closely with aides, Nancy realized that Matthew could no longer solely depend on her for support. His network of supporters had to expand. She knew that she had to step back and allow Matthew to be as independent as possible: “I tried to start letting go in grade school. I knew then that the rest of his life I would be working on turning as much of his life over to him as possible”. This has been a constant struggle. Both Nancy and Tom recalled how they constantly fought the urge to do things for him. As Tom stated, “If he was having a problem with his homework or something, it would have been so much easier for me to step in and just do it, but that doesn’t help him”. Even when Matthew began living on his own, Nancy struggled to not control his life, “I’m so focused on trying to get him to be as independent and as able to manage his life as possible. I just try to stay out of things. It’s really hard for someone who’s kind of controlling by nature anyway. It’s difficult to not engineer his life”. Nancy also talked about how others make this hard on her by coming to her about all things that have to do with Matthew: “People turn to me because they know I know him so well. It’s almost impossible not to have me involved in things because I’m there and I know what to do. I know what his weaknesses are and his strengths and he trusts me”. Nancy described how hard it was on her to tell people that she could not always be the person they go to when something is going on with Matthew, “I just have to keep out of it. I have to back off and let whatever happens happen or else they will always count on me, and what will happen when I am gone?” This continues to remain a challenge in their lives. Nonetheless, both Nancy and Tom are determined to assist Matthew in being as independent and self-sufficient as possible.

Lynn was very upfront about how important Tyler’s independence was to her since his infancy. She explained that when Tyler was a baby she never put him in a playpen or anything else that might restrict him from exploring his environment. Lynn was determined to have this remain her philosophy for raising Tyler. Once Tyler was diagnosed with autism, his support became Lynn’s major focus in life. When Tyler was 4-years-old, the family sold the large home they had just built and moved closer to the city so that Tyler could receive services: “We made a decision. We decided that it was more important for us to have Tyler than to have a beautiful house”. For the next few years, Lynn provided Tyler with a strong foundation of support by developing a close and loving relationship with him. As he entered grade school, Lynn realized that she had to begin to give more control to Tyler so that he would not become overly dependent on her, something that she feared. Lynn talked about being torn between becoming too close to Tyler and letting him be independent:

When I was isolated with Tyler so much it created an incredible opportunity for intimacy and connection, but at the same time it also can move in a negative way in that it can move toward enmeshment where you can’t seem to do or be anything outside of each other. That’s dependence. I had to figure out how to find myself and how to help him find himself away from me. I knew when he was in 6th grade I needed to figure out how we could start separating. You create a strong supportive and loving relationship in those early years, and then there’s the separation that has to happen. I knew for my own survival and for his that we needed to start separating because we were so intimately involved in each other’s lives.

In order to avoid becoming enmeshed, as well as for financial reasons, Tyler moved into a group home in the same neighborhood as Lynn. Although this change was extremely difficult on both of them, Lynn felt it was the best way for Tyler to continue his process towards independence. She worked hard on making sure Tyler was making all the choices in his life, “I’ve tried to help foster him making his own choices as much as he can. Sometimes I might come in with my own agenda and then I have to watch it. I have to be very conscious of that happening and let him have the final word”. Lynn and Tyler both feel that they have been able to develop independent identities. Lynn is still very active in Tyler’s support and advocacy.

#### 3.3.2. Presuming Competence

Participants with ASD identified that it is essential for their supporters to presume their competence. Although it was very important to them that others knew they were not intellectually disabled, they felt it was equally important that others also assumed their personhood. Participants shared a common desire to be treated like a regular person–a person with thoughts, emotions, a sense of humor, and a personality. Tyler focused on how important it was for his supporters to “talk to him like a real person”. Matthew talked about “feeling challenged” when people did not assume he was an intelligent and capable person. Matthew further explained:

Some autistic people are also retarded, but I am not. A lot of kids in my classes thought I was retarded because I looked and acted kind of weird. I have trouble communicating, but I am very smart. My non-verbal IQ score tested at 144 when I was 14. When I took a test of visual/spatial skills when I was in junior high, I scored higher than the top of the high school scale. My parents haven’t even been able to understand my math homework since I was in the 5th grade. I worked very hard in school. I have always done my own homework in all my classes without help from my parents.

For Sue, it is very important that supporters understand that she has her own personality and sense of humor. She also described how hard it was for her to show all her competencies:

It is extremely difficult to explain to someone that I have normal intelligence though I look as if I am disabled. Many do not understand that my intellectual functioning is far greater than is perceived by looking at me. I have a difficult time communicating with the outside world because other than echolalia and verbal prompting I am very limited in my oral speech. I am a junior in college and have a GPA of 3.67. I am not aided in test taking or writing or essays, my college work is my own, contradictory to what many perceive when they view me and my staff in my classes. I do have an aide that takes my notes in classes and that is there for emotional support. Other than that, I am the one responsible for the grades that will appear on my semester grades. Things are not always what they seem. I sometimes feel as if I am the eighth wonder of the world as people stare and marvel at my irregular behaviors which lead to poor assumptions that I am simply mentally disabled with little or no intellectual functioning. My appearance is very deceptive, and day after day I am working, as an advocate for all autistic individuals, to let the world know that we are intelligent and witty, should not be judged for our quirky behaviors because they are only a minute reflection of our true capabilities.

[34] (p. 95)

These individuals felt that the people they identified as significant supporters presumed their competence. They explained that having their supporter believe in them was one of the most powerful supports they could receive. As Stephen explained, “It really helps me when people believe in my abilities”. Tyler described how his Mom always knew that he was intelligent and capable: “She always knew I was there intellectually”. Lynn recalled how Tyler would spell out words he saw on Sesame Street with his magnetic letters as early as a year old. Liz also talked about appreciating and understanding Stephen’s intelligence, “He was just always very precocious. From a young age we realized how smart he was”. When Stephen was able to program a computer at the age of four, Liz realized her son had many intellectual gifts.

#### 3.3.3. Understanding

In addition to presuming competence, participants with ASD explained how understanding who they were as a person was critical to their support. Sue talked about supporters needing to find a balance between understanding her skills and also understanding the impact that autism had on her life, “It is very important that the support understands my intelligence and my autism”. However, participants talked about wanting supporters to know them, not just autism. In fact, some participants with autism talked about wanting supporters who knew little about autism. They recalled negative experiences with supporters who never took the time to get to know who they really were because they assumed everyone with autism was the same or fit some description they had read in a book. Because of this, Tyler tries to avoid hiring staff with a background in special education or autism. He prefers to teach them all they need to know about understanding him. Janna agreed:

I prefer to have people without any experience. People have come in with a special education background and they have all these misconceptions such as, ‘people with autism have no feelings.’ You know I don’t want to hear that from people. I would much prefer somebody to show up and say, ‘I don’t know a damn thing about autism. I would say, ‘That’s great. Tyler and I will teach you all about that.’

Janna also felt that staff who knew little about autism were more willing to spend the time to get to know Tyler and not assume they knew him because he had the label of autism.

Supporters described how they felt they were not necessarily experts in ASD; rather they had a deep understanding of the person they supported. Aishling, Lisanne, and Emily all spoke about how numerous people have wrongly assumed that they were experts in ASD. Lisanne talked about her experience of being approached by people who viewed her as an expert:

At conferences people always say to me, ‘What can we do to get you to work with this person or that person? Or come in and do a workshop or something.’ It’s not that we are experts in autism. We just have a great relationship and we understand Sue. She respects us, we respect her, and you can make things happen when you have all those elements. We weren’t just supporting a person with autism; we were supporting Sue.

Lisanne and Aishling felt that that “autistic” was just one of many characteristics that Sue possessed, but it certainly was not the only one. They also emphasized that knowing what ASD was did not mean you knew who Sue was.

Stephen reported that he gets upset when people assume too much about him without trying to get to know him. Although Stephen likes being labeled “high-functioning”, he felt that the label caused people to overlook some of his major challenges. He found it hard to convince others that he needed help in certain aspects of his life, especially social skills:

People need to understand that there are people out there that haven’t had the same experiences they’ve had and their set of knowledge is going to be very different and things are going to be new to them. No one taught me how to make friends. People aren’t born knowing this. It took me a long time to get people to teach me social skills. In high school, my mom and I arranged for them to teach me social skills–finally! We arranged for me to go to things like football games and prom and homecoming and things like that. I had never really known what to do at those types of things, so I would never really go to any extra curricular things for most of my life. One of the things I want to point out is that I don’t want another child to grow up not having any friends or not knowing how to make friends. That is something they literally had to teach me from step one. And it seems very sad, and it is.

#### 3.3.4. Inclusion

Being included within society, including family life, social situations, and schools, was identified by individuals with ASD as an essential property of their support. The participants with ASD have each been included within society in varying degrees throughout their lives. A common theme among these participants was that they were all included within their families as an equal member. Rita explained how Sue had always been included, even when her family thought she was retarded. No matter what the activity was, they always found a way to include Sue. Rita also made sure that Sue experienced typical activities when she was a child. Rita would not use Sue’s behavior as an excuse to not include her. Rita talked about training Sue so that she could be included in activities such as going out for dinner with the family:

For years Bob or I trained her to sit in a restaurant properly. She could not yell or grab people’s food. We didn’t tolerate behavior that would be upsetting to other people and so she learned–it took time–that she had to behave when she was in a public place.

For Rita, the thought never occurred to her to not include Sue within the family. Sue commented on how her parents have always included her, even before they realized she was not retarded, in her documentary *Autism is a World* [33]:

When I wasn’t able to communicate, actually I was a non-person, yet I was always treated well. Everyone in my family and at school were great at including me. Socially, intellectually, culturally and personally, I have been the most blessed with parents who support me.

Sue described how her friend’s and family’s willingness to include her in their lives has enabled her to become more social:

On of my greatest goals is to become more social. This is slowly but surely being achieved with my core group, which surrounds me. They keep me social by bringing me out into new environments, an undertaking which I would never have imagined possible before I met them. They are my friends, which means for the first time in my life I am able to meet others through them. I go to parties with them and their friends, which I now can consider mine. I have never been happier.

[34] (p. 89)

Every participant with ASD, to varying degrees, has experienced inclusive education. As well, each parent in this study has fought and advocated for their child to be included within regular education. Their stories are much too involved to be included here. However, parents did mention that fighting for inclusion was something they just felt was right, something they felt deep down in their gut, even though almost everyone in their lives was trying to convince them otherwise. Liz explained the importance of going with her gut feelings:

People need to tell parents that they need to go with their guts. The whole time that I did everything against what people told me it turned out right-even though I am a very logical person and I do research and I do all these things. All my life I like to go with a feeling inside. I don’t know how to explain it–a comfort level inside. And if it doesn’t feel right then I don’t care what anyone says, I’m not going to do it.

Both participants with ASD and their supporters agreed that being supported within inclusive environments allowed the participants with autism to experience many things that normally would not be available to them and that this made a huge difference in their lives. Pat described the effectiveness of supporting Peyton in an inclusive education environment, “It was a very normal situation as long as the support was in place. The support wasn’t normal, but in place it allowed Peyton to function and get that normal experience”.

#### 3.3.5. Communication

Both participants with ASD and their supporters identified communication as one of the most essential properties of their relationship. Tyler described it as his most “critical need” and said, “communication is the foundation to my success”. Sue talked about how her ability to communicate changed her life:

It wasn’t until I was able to communicate that I became a part of society. Now I could actually participate in classes, be a friend to people who wanted to extend friendship to me, actually enjoy cultural events such as concerts and museums, and assert my wishes as to where I want to be and what I want to do. I am now a person rather than a non-person.

[35] (pp. 418–419)

While exploring these supportive relationships, we realized that the communication issues for these individuals were very different and required very different supports, especially between individuals who spoke and individuals who used an augmentative or alternative form of communication, such as facilitated communication. Therefore, I will discuss communication for participants who used speech and participants who used augmentative and alternative communication (AAC) in two categories.

Matthew and Stephen were the two participants in this study who used speech as their primary form of communication. However, communication was still a major challenge in their lives. While interviewing Matthew and Stephen, we noticed they struggled to communicate with us, especially about personal topics such as relationships. As Matthew stated, “Communication is challenging for me”. The individuals who support Matthew and Stephen also spoke about how communication was the most challenging aspect of supporting them. As Claire noted, “I think communication is the most challenging thing for him because of how social it is. I mean he knows all the words and what they mean, but putting them together socially is really hard for him”. Nonetheless, all supporters realized how essential it was to work on communicating with Matthew and Stephen in order to develop relationships and to learn how to better support them. They described numerous support strategies that they have developed to work on communication.

Abby explained how she developed strategies to help Matthew communicate with her. She talked about always giving him a time frame “so that he knew how long the conversation would last”. She also talked about keeping communication “simple, short, and concise”. Abby would also support Matthew by giving him extra time to respond. Abby recognized that communication was very hard for Matthew and that he often said, “I don’t know” or “yes” when he was really just trying to get out of the conversation. Over time, as Abby started to develop a relationship with Matthew, she noticed that he was much more honest and open with her. Upon reflection, she felt he had to first develop that relationship and gain trust before he was comfortable communicating with her. Nancy talked about supporting Matthew with his communication by always checking for understanding through questions. For example, Nancy would ask him, “Is this what you meant when you said that?” She would also ask questions to ensure that Matthew understood a message from someone else, “Matthew, what did that lady tell you that you needed to do?”

Claire and Deborah talked about the importance of having open and honest communication with Stephen. Claire stated, “You have to tell him things straight out because sometimes things just don’t occur to him. I think communication is the most difficult piece for Stephen, especially in social situations”. They gave many examples where support was much more successful for him when they were open and direct with their communication. Deborah explained how she was open and honest in her communication with Stephen and always checked for understanding: “We talk about anything and everything and we hit it right on the nose. We don’t dance around anything. He very much wants that. We don’t down play any of the disabilities he has and we make sure he understands”. She also described how she works with Stephen’s “body movements, vocal out put, intonation, and giving people the opportunity to talk”. Liz realized that Stephen, though very verbal, had challenges communicating and understanding communication. She recalled how she would spend a lot of extra time explaining things to him: “I remember when he had to learn Shakespeare. We must have watched Romeo and Juliet one hundred times. We went over every single thing, but eventually it paid off; he began to see what was happening”. Although the challenges that Stephen and Matthew faced were distinctly different from participants who used AAC, communication still remained an important element of their support.

For Peyton, Sue, and Tyler, communication requires the support of another person. Peyton, Sue, and Tyler, as well as their supporters, explained the essential elements required for their communication to be successfully supported. These included: recognition of their ability to communicate, having the desire to communicate with them, developing a relationship, and constantly seeking understanding.

Peyton, Sue, and Tyler talked about the importance of their supporters recognizing and believing in their ability to communicate. Tyler explained that this required supporters to “look beyond my outward appearance and give me a chance to show you that I can communicate”. Tyler explained how this required time and energy that many supporters were not willing to provide. However, he felt blessed that Janna and Lynn both were willing to make that effort. He described how Janna had always believed in his ability to communicate, even when his communication techniques made him look like a “car wreck”.

Sue also explained that Rita was relentless in her drive to support Sue’s ability to communicate through typing: “Rita demanded that I communicate through facilitated communication, not behaviors. Actually that forces me to fight my killer autism and think. Rita and I have communicated tremendously over the years to build the right support for me”.

In order to support Sue, Tyler, and Peyton, others must have the desire to want to communicate with them, as all three often have difficulty initiating communication. Aishling explained how important the desire to communicate with Sue is in order to really know her, “You’ve got to want to know. If you don’t care to know, you’re not going to know. I feel bad when I hear that she has support staff who don’t type with her. They don’t get it. They’re not getting the whole Sue”. Participants with ASD wrote about how the supporters they identified do have that desire to communicate with them. For example, Tyler talked about his mom, Lynn, always wanting to “hear my voice”.

Supporters described how they constantly sought opportunities for communication. Rita and Emily both talked about how this is a major priority for them. Both of them tried to put themselves in Sue’s position and realized that they would want to have as many opportunities to communicate as possible. Rita described how she constantly gave opportunities for Sue to communicate:

From the time she started typing I would always ask her many times throughout the day, ‘What do you think about this?’ Or offer her opportunities to talk about something. I would always go to her right before she goes to sleep because I thought to myself if you’re not able to communicate all you want throughout the day then there must be stuff that at the end of the day you want to talk about or that’s on your mind when you are ready to go to sleep.

Rita went on to describe how Sue always had something she wanted to say; this became a very special part of the day for both of them. Emily also described how she gives Sue as many opportunities to communication as she can:

Throughout the day I try to give her opportunities to talk just like any other person might want. I know she has a lot to say. And I try to give her opportunities to talk about everything, not just stuff about support and school. I’m so into her life, we talk about all sorts of things and that’s important too. It helps us maintain a friendship.

Because their opportunities to communicate are dependent on another person, participants described that it is essential to have a trusting relationship with that person. Tyler talked about the relationship being the “foundation of facilitated communication”. He explained how he needed to build a loving relationship with the person who facilitated his typing, “I need that foundation so I can focus on my communication. I need unconditional love and respect”. From my observations of Janna facilitating typing with Tyler, it was very obvious that he had found that relationship with her. Aishling could not ever imagine trying to support Sue without having a relationship with her. She talked about the strong connection between relationship, support, and communication: “You can’t have a relationship without communication and you can’t support without a relationship”.

It was very important to these individuals that others understood their communication. This was often a challenge for participants with autism because they reported that their bodies, voices, and facial expressions were often unreliable forms of communication. Sue explained how she demands that her staff communicate with her through facilitated communication because her voice and behaviors are not reliable: “My behavior actually contradicts my thoughts. It really is really vital that I communicate”. Although Tyler is able to read aloud everything he types, he talked about how his spontaneous speech “still sucks” and that it was crucial that supporters type with him so that his communication is clear and reliable.

Understanding Peyton’s communication, whose typing was more cryptic and poetic than the other participants, is a constant challenge for the people in her life. Martha talked about how she supports Peyton with communication in a much different way than she does other friends: I need to give her more communication support than I would give to most of my friends. I need to dig deeper more often for meaning than I do with other friends. I need to accept that she’s doing her best more frequently than I do with some other friends. And I need to offer other possibilities for her to communicate.

Martha further described how she constantly seeks understanding of Peyton’s communication and behaviors: “I am always on the lookout for meaning and if I make mistakes, which I’m sure I do, I’m more likely to err on the side of making an assumption that she didn’t intend to communicate”. Martha takes many things into consideration when she is seeking meaning from Peyton’s communication, “I look at the context of what our conversation was or is, the timing of her response, and other things such as her eye to eye gaze, her positioning, and her affect. And in the end it’s my best guess”. Martha does check with Peyton to determine if her “guess” is accurate, “I always check with her. She capable of objecting and I tell her she can always tell me otherwise”.

#### 3.3.6. Collaboration

Although it was important that the participants with ASD have control over their lives and support, they also stressed that supporting them involved collaboration. In many instances they described not knowing exactly what kind of support they needed. They reported needing input and insight from the people around them. As well, supporters described how they are not always sure how to provide support and feel they need feedback from the individual. The support that took place within these relationships involved a great deal of collaboration and teamwork. Tyler and Janna described the “constant dialogue” that took place around support. Tyler explained how they “talk a lot and figure things out together”. Janna described it as an “agreement or negotiation where Tyler always has the final say” and her suggestions for support are always centered on his needs:

We talk about support together, but it is always his choice. I always ask him first, ‘How do you think we ought to approach this or what do you think we should do? I’ll do whatever you think is important? What do you want me to do?’ Sometimes he doesn’t know and I tell him what I see us doing and we talk about that.

Sue and Emily described collaboration in a similar way. Emily described how Sue and her staff view her support as a “team effort”, which involves constant communication not only between staff and Sue, but also among staff:

Sometimes she doesn’t know what would be best for her but a lot of the time she does–if you just ask her. She can tell you, ‘that wouldn’t help me’ or ‘that will.’ All the staff has to really communicate with her and with all the other staff. A lot of the time we talk through it with her and sometimes she doesn’t know if something will work or not and we don’t either. But we talk about it and try things out. Sometimes it works, sometimes it doesn’t.

For all participants, support was a constant negotiation that required both the supporter and individual to work together as a team. Support providers did not need to have all the answers, nor did the individuals, but they both were willing to work together at figuring it out.

#### 3.3.7. Consistency and Flexibility

Participants with ASD described needing consistency in two ways: consistency of supporters and consistency of support. Consistency of supporters was something that provided these individuals with a sense of “comfort and grounding”. Changes in staff could be very disruptive. Tyler talked about how changes in staff “scare the hell” out of him and he “prays like hell” that it will soon get better. Sue’s documentary, *Autism is a World* [33], captured the transition of Aishling and Lisanne leaving as support staff. Although they are still Sue’s best friends, the transition was devastating for Sue. The documentary captured Sue’s intense emotional struggle with their leaving. During the film, Sue typed to Aishling that she did not want her life to “be in a hell because she was leaving”. Sue commented that when supporters or staff do leave, she feels torn between being happy for the next chapter in that person’s life and losing a great support: “It is extremely hard to not want to really be happy for the staff who is moving forward with their life, but they are such important assets to me. One of the incredible things that happens is seeing which ones still are really friends”.

Matthew explained that having Abby as a consistent supporter throughout college was “comforting” to him. Abby supported Matthew each year that he attended college, and Matthew believed this consistency “aided his success”. Sue and Tyler also talked about how important consistency of supporters is when they are typing. Both Sue and Tyler described how difficult it was to type with multiple people in the past. Sue described how this inconsistency with supporters was challenging for her in high school:

I really don’t believe I had the right kind of support. The special education staff thought I should type with as many people as possible so I wouldn’t become dependent on one person. However, with a different support person each period of the day, I was not able to type really well with most of them. I could type social conversations but couldn’t do difficult academic work. It was not that I needed them to do the work for me because I could actually type things independently at home but not type the same things with a facilitator at school. I think I should have had two facilitators at school and have had them over a few years. As it was, I had to start each year with several new facilitators.

If consistency of supporters were in place, one would assume that the support would also be consistent. For a lot of participants this was the case; some, however, reported that this was not always true. Sue described needing consistency in the support she received. She specifically requested that her supporters be “firm, consistent, and fair” with their support. For the most part, Sue felt that her supporters were consistent, and when they were not, she would have to remind them that this is essential for her to be successfully supported. Sue also reported that this process became more difficult when the people who supported her were close friends. For example, Sue described how she had to remind Emily of the way she had requested to be supported when she sensed that Emily was feeling that she was being too hard on Sue.

Most participants with ASD agreed that they liked consistent and structured support but realized that their support needs were constantly changing. Therefore, support had to also be dynamic and flexible. Each participant attends or has attended college and presents at conferences. Activities like these require flexible supports. Sue talked about her staff helping her ease away from her dependence on structure and routine:

My staff are my biggest reason routine is not as pivotal in my life anymore. I will admit things are done loosely based on a structure or routine, yet my staff have been able to teach me that things in life are not predictable and that is ok, as long as I am willing to be patient.

[34] (p. 102)

Sue went on to discuss how staff cannot structure every aspect of her life, especially college, where holidays or schedule changes for finals are outside of their control.

For Peyton, recent health issues have forced her supporters to change the way they support her. The range of supports that she requires have varied a great deal, from supporting her to present her valedictorian speech to supporting her to feed herself and go to the bathroom. Support has not been a linear process for Peyton; she requires different types of support each day. Pat described the changing nature of Peyton’s support: “I mean support really changes. It depends on what’s going on with her. It’s so complex. It’s a huge complex issue of even knowing how to talk about it or qualify it”. In order to support Peyton successfully, Pat and Dianne agree that support has to remain flexible. Pat stated, “Support is never going to stay the same. Support has to change because Peyton changes. It would be comfortable and safe to always keep support the same, but that’s not what she wants or needs”.

#### 3.3.8. Personal Characteristics and Interaction Styles

When participants discussed the characteristics they look for in support staff and the styles of support they prefer, it was amazing how these characteristics captured the characteristics and styles of support of the individuals they identified for this study. This section will describe the major characteristics and styles of support that were discussed. Although there were many similarities among the identified characteristics, there were also differences.

Two of the five participants with ASD felt neither age nor sex was an important characteristic in their supporters. However, for Sue and Peyton age was an important factor. Peyton explained how she preferred supporters who were older than she was. Although specific ages were not important, Peyton felt that she worked better with supporters who have had more life experience. Pat observed:

What seems to be the difference is that the individuals we’re talking about have experienced life, they can put it in perspective, they understand that life can be difficult and is difficult and because of these experiences they have a wisdom and because of the wisdom they have a compassion.

Therefore, it was not surprising that all of the supporters whom Peyton identified as significant supports were at least 15 years older than she.

On the other hand, Sue prefers staff her own age. She enjoys being with her peers, and having supporters her age is a way for her to develop relationships with peers. Aishling, Lisanne, and Emily are all very close to Sue’s age and have each developed a very special relationship with her that will continue long after their paid positions. None of the participants talked about the sex of their supporters being an important factor. However, it is notable that of the 17 supporters who were identified, only 2 were male (both fathers).

Participants with ASD talked about wanting supporters who were “kind, loving, and patient people”. Tyler said, “I need to see that person’s heart. I need to know they are a loving soul”. Honesty, integrity, a sense of humor, and a strong spiritual life were also important to Tyler. Peyton described a kind of “purity” that she looked for in supporters. She described this as knowing they had a “helium heart”, one full of compassion and love. Matthew and Stephen also mentioned that it was important to have supporters who were patient, kind, and respectful.

An important characteristic identified by both types of participants was being open to a having a relationship with the person they supported. This openness also involved a willingness to learn and grow with that person. Although this was something that was easily identifiable in each supporter in this study, Janna explained that this was not a common characteristic for many people who attempt to support individuals with autism. She explained, “If people could see the kind of potential relationship they could have with him. If they could hold that vision they would stay forever because he’s just the greatest guy”. Participants also talked about having an openness to learn and to be willing to change any misconceptions they may have about autism. Peyton discussed how it was hard on her to be supported by people who were “overly judgmental”. Peyton felt that this precluded her from being herself, and it precluded her supporters from understanding her.

Sue described wanting supporters who were “firm, consistent, and fair”. She also explained that she wanted supporters to be very “strong-willed”, meaning that she wants supporters who will stand up to her, make demands of her, and push her towards her goals. Sue herself is very strong-willed and she needs someone who is willing to redirect her when she needs it. Aishling, Lisanne, and Emily all talked about how these characteristics were requirements for supporting Sue. Sue has specifically asked them to be very firm and strict with her because that is what Sue feels works best for her. They all described how others perceived of the support they give Sue as being “too firm”. For example, Aishling and Lisanne talked about how others remarked that they “looked like bitches” in Sue’s documentary *Autism is a World.* Although this is only one aspect of their relationship, it was very important to them that I understand that this is what Sue has asked of them. Emily further explained, “Sue is the one who wants it that way. She needs someone on her all the time. So we support her firmly and dictate the flow. We know what works. Sue will walk all over you if you are not tough”.

Peyton described wanting supporters who supported her in a very “determined and relentless way”. She wants supporters who felt that “failure was not an option”. Like Sue, she wants supporters who will not give up on her and will continue under the worst of circumstances.

### 3.4. Future Hopes and Fears

At the end of each interview, we asked participants to talk about their hopes and fears for the future of the individual they supported. Without exception, every participant described as their greatest hope that the individual will develop more deep and substantial personal relationships and increase their network of supports. Their greatest fears were that the individual would not continue developing relationships and would not have people supporting them with understanding, love, and respect. Participants also mentioned hopes about advocacy, future careers, and building skills that led to more independence, yet it was very clear that both their greatest hopes and their biggest fears focused on relationships.

Emily, Aishling, and Lisanne expressed their greatest hope was that Sue would be able to develop relationships similar to the deep friendships that they share with her. As Lisanne stated:

The challenge for Sue is finding more of those relationships. For us, we are able to continue developing relationships and it’s pretty easy for us. But for Sue I want her to be able to do that so that she can grow emotionally and socially. She’s got us. We’re here. We’re not going anywhere. But she needs more of that.

This was also a hope for Sue, “I hope to keep finding awesome peers to support me through college and beyond”.

Abby hopes that Matthew will develop more peer friendships. She realized that Matthew liked to do things with his mother or enjoyed activities by himself. She just hopes that his relationships will expand from there: “I think he would appreciate someone willing to hang out with him who’s not his Mom or his sister or paid support staff. He needs a peer. He does like to be by himself, but every now and then everybody needs somebody”.

Claire and Deborah both mentioned that they hope Stephen will find a companion. Stephen mentioned that one day he would like to get married when he finds the right person. His supporters hope this will happen for him. As Claire stated, “That’s the only thing I ever worry about him–Will he find that companionship?” This is also a hope that Janna has for Tyler, “I know he wants that intimacy and closeness and so I want him to have that. With the right person, I think they could be an amazing couple”.

Parents also reported that they hope their children develop more personal relationships, but their primary concern was insuring that there are people in their lives who will continue to support them after they pass away. Rita spoke about this:

What I would like as Sue gets older is to continue increasing the circle of support that surrounds her. If things continue the way things are going now I think Sue will have a wonderful group of individuals who will continue to support her after Bob and I die. People work with her for a few years and they move on, but they don’t leave her. They stay in touch and stay apart of her life. I think that is the most important aspect–they stay in her life.

Other parents did not seem as confident in securing future supports for their children. This is something that Pat and Dianne really struggle with. As Pat explained, “I’m 64 and Peyton knows that there’s a certain amount of time left and then she doesn’t know what’s going to happen to herself. We don’t have a plan if something happens, but we have to hope that there is a way to provide for Peyton’s future”. Lynn also worries about Tyler’s future support, especially after the sexual abuse incident, “My hope is that he’s going to build a network of support people that are going to love him and be there for him when he needs help and that he won’t be isolated and alone without any form of communication. Those are my greatest fears”. Although parents and supporters do hope that the people they support will finish college or find a career that they are happy with and are respected in, building relationships and increasing their circle of supports remain their greatest hopes.

## 4. Discussion

The purpose of this study was to develop a substantive grounded theory about supportive relationships in the lives of individuals with autism. Additional purposes included: (1) documenting the experience of individuals with autism who are “academically successful” and exploring aspects of their experience with social support that have enhanced or limited that experience; (2) exploring whether and how the mode of communication influences the supportive relationship; and (3) exploring the qualities and dimensions of the relationship. This chapter will be divided into three sections. The first will focus on the findings and substantive grounded theory that emerged from the data. The second will describe the limitations of this study. The final section will discuss the implications of this study for practice and research.

### 4.1. Dynamic Model of Supportive Relationships

**Figure 1:**
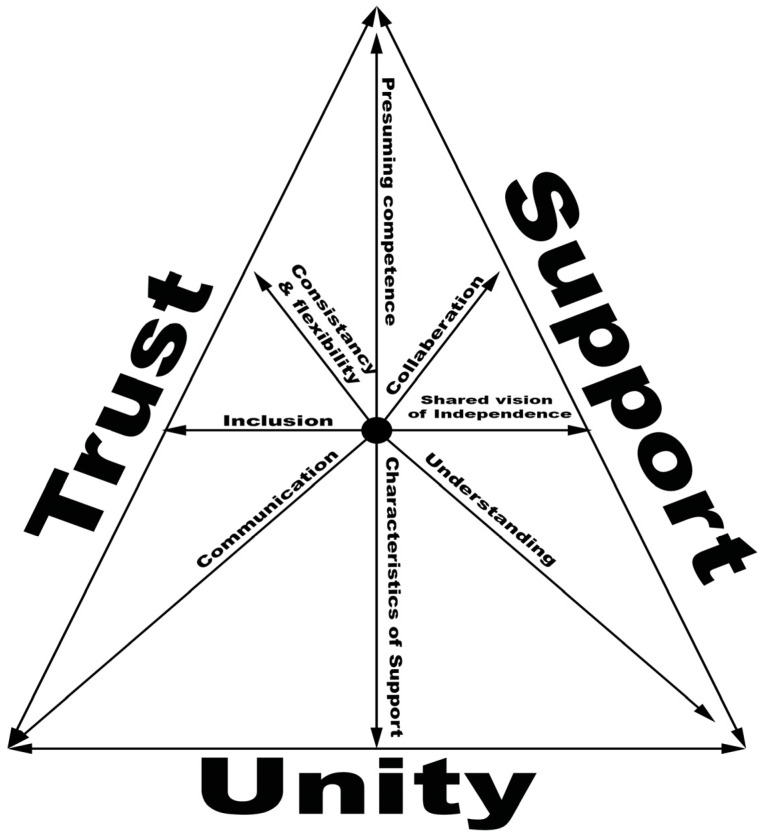
Dynamic model of supportive relationships.

Three core categories emerged as essential to these supportive relationships: trust, unity, and support (as reported in greater detail in [32]). Eight properties also emerged as essential conditions of successful support: (1) shared vision of independence; (2) presuming competence; (3) understanding; (4) inclusion; (5) communication; (6) collaboration; (7) consistency and flexibility; and (8) personal characteristics and interaction styles. Within the supportive relationships that we explored, these categories and properties interacted in a dynamic way; they influenced and interacted with each other in a non-linear manner. Figure 1 represents the dynamic nature of the characteristics of supportive relationships described in this study.

We did not undertake this study with the assumption that a dynamic model would emerge from the data. In fact, although we were familiar with general systems theory, it took a while to “see” the dynamic nature of these relationships. During the data analysis stage and throughout the first drafts of writing, we worked hard at attempting to fit the categories into a linear model. We began with trust as the foundation, drew an arrow up to unity, and then drew an arrow up to support. Was this the process that emerged from these relationships? In order to test this model, we examined how effectively it described each dyad. We started to draw lines that represented each dyad, and when we were done we saw lines all over the page, lines going back and forth, and lines starting at different points. It was at that moment that we realized we were looking at this process in the wrong way. We had conceptualized processes or trajectories as linear lines starting at one point and moving towards the next. We were having trouble “letting go” of stage-theory developmental models, which appear to fit complex processes into clean, linear models. Once we saw all the confusing lines running through our linear model, we realized that the process of supportive relationships in the lives of these individuals was a non-linear process; it was complex and dynamic.

Hill and Leary [36] described dynamic systems as consisting of collections of related sub-systems that are usually viewed as a single entity. Fogel [37] provided additional characteristics of dynamic systems. First, systems are complex and involve interdependent parts. Changes in any single part of the system results in “corresponding changes in other related parts of the system” [37] (p. 46). Second, systems are organized, meaning that the system can be described as a single entity independent of its parts. Third, systems are self-stabilizing and self-organizing. Fogel stated: “The collective properties of the organization are generally stable tendencies maintained over time by the transactions of the individuals and their relationships” [37] (p. 47). The stability of the system is maintained through “dynamic fluctuations of activity between its component parts” [37] (p. 47). Fourth, systems exhibit equifinality, meaning that different dynamic processes can lead to similar systems. Lastly, systems form hierarchical patterns. The system may include higher or lower orders within the same model, yet “all orders are part of the same system and are the natural result of the system’s dynamics” [37] (p. 47).

The following points highlight how the supportive relationships explored in this study are dynamic systems. First, supportive relationships are complex and involve interdependent parts. The findings revealed complex relationships that involved three core categories and eight properties. Changes in any of these resulted in changes in other categories or properties. For example, if trust was violated, support was affected. Additionally, if a shared vision of independence did not exist, support was affected as well. Second, supportive relationships are organized. Participants were able to discuss their relationships as whole systems and as separate components. Third, supportive relationships are self-stabilizing and self-organizing. The relationship is stabilized through the maintenance of each property. For example, support is only successful when both trust and unity are maintained. In order for the relationship to remain trusting, unified, and supportive, all categories and properties within those categories must be constantly maintained. Fourth, supporting relationships exhibit equifinality. The findings indicated that supportive relationships could develop in a variety of ways, such as through friendships or paid staff positions, and include individuals with a variety of personal characteristics and backgrounds while still sharing a common outcome of successful support. Lastly, supportive relationships consist of hierarchical patterns, higher or lower orders within the same model that all play a part in the system. Three core categories and eight properties emerged as essential conditions of these supportive relationships. Although participants identified the core categories as the most significant aspects of the supportive relationship, the properties were also essential to its development and maintenance.

### 4.2. Trust, Unity, and Support

The substantive grounded theory and the findings of this study suggest that trusting and unified relationships are at the core of providing support to the individuals with ASD in this study. These overall findings share many similarities with the literature on personal relationships and social support within the general population, suggesting that these relationships are very similar to relationships among non-disabled individuals. Additionally, many of the findings of this study questions our current understanding of ASD as well as the diagnostic criteria of autism presented in the *DSM-5* [3]. The following sections will highlight the most significant findings of this study and describe how they relate to the professional literature.

#### 4.2.1. Trust

Veenendall and Feinstein [38], whose research focuses on relationships in the general population, described trust as a universal value that is essential for maintaining an effective and long-lasting relationship. Participants identified trust as the foundation of their unified and supportive relationships. Trust needed to be constantly maintained and tended to by both members of the dyad. Violations of trust were particularly devastating to participants with ASD, affecting both current and future relationships. This is consistent with literature from the fields of personal relationships and social support within the general population; as Leatham and Duck [39] (p. 9) stated: “If previous attempts at support have had negative outcomes, a person may blame the partner, devaluing present support attempts”.

Individuals with ASD described trust as a prerequisite to effective support and, therefore, wanted to feel this trust with their supporters as soon as possible. However, building trust within these relationships took time and effort. Recall that participants with ASD described feeling that they had more at risk than their supporters, primarily because they felt they had to trust the other person to be responsible for their lives. Veenendall and Feinstein [38] explained that trust was difficult to build in any relationship because of the risks involved. A few participants with ASD described testing their support providers to determine if they could trust them. They also described needing to “know” or having a “feeling” that the person who supported them had their best interests in mind and would be there for them in times when support was needed. This was something that the supporters recognized and respected.

These findings are consistent with Bambara et al. [20], who reported that staff members working with individuals with severe challenging behaviors felt that trust was important in their relationships with these individuals. They also reported that trust between staff members and the people they supported took time to develop. However, the findings in this study are not consistent with the professional literature in the field of ASD, particularly the “theory of mind” model. In fact, these findings call into question the “theory of mind” model, which argues that individuals with ASD are unable to understand the thoughts or emotions of another person [40,41,42]. Recall that Tyler stated that he needed to “see the person’s heart” and know that the person was a “loving soul”. Also, Peyton described that sometimes she knows right away that she will not be able to develop a trusting relationship with a staff person. Knowing this requires the ability to read or assess the other person, including the ability to think about another person’s thoughts, feelings, and intentions. Participants also reported that trust must constantly be maintained. This also requires these individuals to constantly monitor and assess the other person’s thoughts, feelings, and intentions. All of the skills mentioned above require a “theory of mind”. These findings clearly indicate that many of the participants with ASD in this study do understand what another person is thinking and feeling, which questions the usefulness and accuracy of the “theory of mind” representation of ASD, at least for these participants.

#### 4.2.2. Unity

In the supportive relationships that we explored, support was given and received within the context of relationships. Leatham and Duck [39] argued that the strongest examples of successful social support within the general population take place within the context of close personal relationships, as opposed to more distant and less personal interactions. Yet, the mere existence of a proximate relationship between the person with ASD and the supporter was not the determining factor of successful support. Rather, it was the quality of that relationship. These relationships all exhibited a similar quality which one participant identified as “unity”. This section will further discuss the properties that constitute a “unified” relationship, as well as describe the trajectories of these relationships. These findings will also be compared with the professional literature.

Properties that define a unified connection according to the participants in this study include: intimacy, mutuality, and reciprocity. Each will be discussed below.

• Intimacy

Snow [43] stated that one of the gifts individuals with disabilities bring to the world is intimacy. Both participants with ASD and their supporters described having a deep and intimate bond with each other. The connection between Janna and Tyler serves as an excellent example. Their relationship is a deep, loving, and intimate bond. This came across in their words and actions. Not only were they affectionate and loving towards each other, they were also connected in a cerebral way, as evidenced in the quickness of both of their wits. Their relationship was only one of the many examples of intimate and unified bonds that emerged in this study.

• Mutuality

The *Oxford English Dictionary Online* defined mutuality as the sharing of or in an emotion, desire, aim; fellow feeling, community; interdependence. A significant finding of this study was discovering how mutual these relationships were. Both members of the dyad shared common beliefs, emotions, desires, and goals. As well, support and affection was a shared activity. Many supporters described instances when the individual with ASD supported them. Recall how Aishling, Lisanne, and Emily described how Sue gives them advice and insight that greatly influences their lives. Also, Janna described numerous instances when Tyler was there for her when she needed a friend’s support. In sum, “Mutuality…allows the possibility of working *with* the other person, not just for them” [44] (p. 221).

• Reciprocity

Reciprocity involves mutual action and influence, implying a give and take aspect to the relationship. However, the give and take that was involved within these relationships was not necessarily similar or equal. What each member of the dyad gave and received was very different. Nonetheless, it was evident that the reciprocal nature of these relationships was the most rewarding aspect of these relationships for the support providers. They described very personal accounts of how these relationships were reciprocal. For example, Lynn, Tyler’s mother, beautifully captured how her relationship with Tyler allowed her to better learn who she was and helped her find her own voice. Also, Aishling described knowing herself better through her relationship with Sue. Martha also explained how Peyton provided her with insights and details about Peyton’s life experiences that Peyton knew she would find interesting. Recalling these accounts was a very emotional experience for many participants. Many supporters felt that they received more in return than they gave. These are only a few examples of the reciprocity that existed within these relationships.

These findings are congruent with Taylor and Bogdan’s description of “accepting relationships”, where non-disabled individuals reported that their relationships with people with disabilities were mutual and reciprocal, even though what they received was qualitatively different than what they gave [45,46]. Also, these findings correspond to studies that described relationships between non-disabled people and individuals with disabilities as intimate, deep, and loving [20,21]. However, these findings question the diagnostic criteria of ASD, which describes individuals with ASD as having an inability to develop and maintain social relationships and lacking social and emotional reciprocity [3].

• Trajectory of Relationships.

Participants described developing relationships in a variety of ways. Nonetheless, the manner in which relationships developed between non-related supporters and participants with ASD did not appear to be a determining factor in the quality of the relationship or the effectiveness of support. Some support participants were first friends and later became paid support staff. For example, Aishling was first a high school friend and later became a paid support for Sue. Others began as paid staff and later developed a close relationship with the individual. For example, Emily described the difficulty she faced when first working with Sue. It took quite awhile for them to become friends. Thus, relationships that first began as friendships and relationships that developed within paid positions appeared to have an equal chance at becoming trusting, unified, and supportive.

Although these relationships did not share similar trajectories, one common theme among non-related supporters was that at one time or another, the support participant was paid to support the individual with autism. Taylor and Bogdan [46] also found that some of the closest relationships were between former staff members who remained friends with the individual after leaving their job. The intimacy involved in these jobs most likely aided in the development of close relationships. Another determining factor might be that several individuals with ASD in this study spent the majority of their time with paid staff.

On the other hand, the trajectories of the relationships between individuals with ASD and their parents did share many similarities. Parents described that they had developed strong and loving bonds with their children during infancy and early childhood. As time went on, parents described turning their focus to their children’s independence. Once their children became older, this emerged as a shared vision, thus unifying their relationship even more. This trajectory appeared between each parent and child dyad explored in this study.

#### 4.2.3. Support

Successful support depended on trusting and unified relationships. Participants with ASD reported that support was most effective when their supporters espoused certain beliefs and took specific actions, which are discussed below.

Support required more than just physical assistance; it required the supporter to believe in and share dreams and goals with the person they supported. The beliefs that participants identified as essential to successful support included presuming competence, understanding, and sharing a vision of independence.

Participants described presuming competence as involving understanding and believing that the individual with autism is a competent and intelligent human being. Nonetheless, both types of participants realized that the person with autism also needed significant support and that presuming *all* competencies was unrealistic and not supportive. For example, when Sue attends classes at college she needs a support person there to take notes for her. She requires these specific supports in order to be successful in college. An equally important support is that her staff understands and acknowledges that Sue is capable of learning and participating in class. However, if they were to presume that Sue could attend class by herself and take her own notes, this assumption would not support Sue. Therefore, presuming competence does not necessarily imply presuming *all* competencies. Rather, it refers to others being open to notice signs of competence. If a supporter assumed that the individual was incompetent, then this would not allow them to be open to noticing signs of competence.

For these participants, presuming competence meant that they were viewed as essentially competent individuals, rather than deviant and deficient, as people with disabilities have been primarily viewed throughout history. Participants felt that they constantly had to prove their intelligence, whereas this is generally not the case for a non-disabled individual. Their greatest desire was to be seen as just a typical person who may need some extra supports and accommodations.

Assuming “personhood” was a critical feature of presuming competence. Individuals with autism in this study desired to be treated like a typical person–as a person with thoughts, emotions, a sense of humor, and a personality. Participants with autism all felt that their supporters included in this study assumed that they were intelligent human beings and that with the right support in place, they could succeed. These types of attitudes were a significant factor to their success. These findings are synonymous with the presuming competence concept presented by Biklen and Cardinal [47] and are similar to Bogdan and Taylor’s [45] work that described assuming “humanness” as a characteristic of accepting relationships.

Participants described the importance of having a deep understanding of each other. Most discussion focused on understanding the labeled individual. Participants with ASD desired to be seen beyond their label and the stereotypes associated with this label. They did not want their supporters to understand “a person with autism”; instead, they wanted them to understand and know Sue, Peyton, Tyler, Stephen, and Matthew. Recall how Aishling explained that ASD is just one of many characteristics that constitute who Sue is–it is not her only characteristic. As Kluth stated: “If you know one person with autism, you know ONE person with autism” [48] (p. 2). This deep understanding is similar to what Kliewer and Biklen [21] described as “local understanding”, which involves supporting individuals with disabilities through deep and intimate involvement. However, these findings question much of the disability literature that tends to describe individuals with disabilities through typological thinking and sweeping generalizations such as “all people with down syndrome are happy” (see also [49,50]).

For participants with ASD, sharing a vision of independence meant that the people in their lives share their dream and goal of independence and support them towards this goal by consistently believing in them. An optimistic, positive, and hopeful mindset was essential. Recall that many participants with autism reported that they could “sense the emotions” of those who supported them. If these individuals could sense positive feeling of encouragement and belief, that could be an amazing emotional support. On the other hand, sensing negative feelings from their supporters could cause the individual to doubt their own capabilities. Again, this reported ability to sense the emotions of others further undermines the theory of mind model.

Sharing a vision of independence also involved letting the individual with autism have control over every aspect of his or her life to the fullest extent possible. A common theme with these individuals was that they *did* have the primary control over the decisions in their lives. Their supporters were there for them in every way they could be but realized that the final say was always in the hands of the individual with autism. Supporters understood that controlling the labeled person was not helpful.

Participants also reported that “over-supporting” did not assist them in their goal of independence. Individuals with autism described wanting to try things first on their own and ask for support only when it was needed. For example, Sue described wanting her staff to push her to be able to do as much as she could with the least amount of support necessary. Therefore, supporters had to constantly walk the thin line between over-presuming competence and over-supporting. Somewhere in the middle was the right amount of support necessary for the individual to move towards independence. Through communication, collaboration, and trial and error, supporters and participants with autism were able to find the amount of support necessary for the success and independence of the person with autism.

Successful support required the combined efforts of both the labeled individual and supporter. Often the actions that would result in effective support were unknown or, if known, difficult to obtain. For example, many parents described battling with school districts to ensure that their children were fully included within the general curriculum. In many ways, participants, both individuals with ASD and their supporters, felt that they had to “pave the path by walking it”.

Participants described particular actions that were required for effective support including: inclusion within schools and the community, supporting communication, promoting and practicing collaboration, and providing consistent and flexible support. Each will be discussed below.

All participants described being included within family life, social situations, schools, and the community as essential for the person with ASD. Although being in these environments often required greater support, only in these situations did participants feel that the person with ASD could learn and grow. Not one of the participants favored segregated, artificial, or highly structured environments that are often offered to individuals with disabilities, particularly ASD.

Leatham and Duck [39] described personal relationships that provide support as being “situated in and given context through communication” (p. 5). Communication was described by participants as one of the most essential properties of their relationships and support. Participants also identified that they needed the most support with communication. In fact, supporting individuals with their communication needs was one of the most time consuming activities of the day for many of these participants. Yet, all participants recognized its necessity. Although communication was challenging for both individuals who used speech as their primary form of communication and individuals who used facilitated communication, all supporters constantly sought opportunities for these individuals to communicate, which went far beyond just asking them to make simple choices. The goal was for the individual to have as many opportunities to communicate as any other non-labeled individual would have. Although communication required a variety of supports for each participant, the quantity and quality of communication was very similar to non-labeled individuals.

Supporting individuals with their efforts to communicate involved more than providing opportunities to communicate. It also included spending considerable amounts of time understanding the meaning and intent of communication and supporting individuals who use alternative or augmentative forms of communication, such as facilitated communication. Recall how Sue described that her voice and behaviors were often misleading forms of communication. For example, if a supporter asked Sue a “yes” or “no” question and she responded verbally “yes”, that did not necessarily imply that she meant it. It may have been that she got stuck on the word “yes” and, therefore, verbally repeated it. Supporters had to be committed to constantly seek understanding of Sue’s communication, which involved looking beyond her echolalic speech and behaviors. Sue identified facilitated communication as her only reliable and accurate way to communicate. Leary & Hill [51] described that when communication is challenging for an individual “it becomes necessary to suspend absolute trust in one’s intuitive interpretation” (p. 44) and assumptions about meaning.

Effective support also required promoting and practicing collaboration, along with providing consistency and flexibility. The specifics of support were something that participants felt had to be worked out collaboratively between the support participant and the individual with ASD. Supporting these individuals was not about power, control, or authority. Support was a joint effort with each member contributing. Also, support had to be both consistent and flexible. Consistency of supporters and support was comforting to individuals with ASD. On the other hand, they realized that support also had to be flexible due to changes in the environment that were outside of their control.

The beliefs and actions discussed above were identified by all participants as the essential conditions of support and were forefront in the support that these individuals received. They are also similar to many strategies promoted by the natural supports literature [52,53,54,55,56,57,58].

### 4.3. Limitations

There were notable limitations to this study. This discussion will be divided into two sections: limitations concerning participants and limitations concerning data collection and analysis.

This study included a total of 22 participants, only five of whom had the label of autism. A small sample places some limits the ability to generalize the findings to other individuals with ASD. Likewise, the selection criterion of being “academically successful” greatly decreased the population from which I could make a selection. Also, the participants in this study were all individuals with ASD who were able to communicate through either verbal language or traditional orthography. Many individuals with autism are very limited in their ability to communicate their thoughts.

As some of our criteria were limiting, we sought other ways to promote variety among my participants with ASD. We specifically sought out male and females, as well as individuals with ASD who used speech as their primary means of communication, along with individuals who used an augmentative and alternative form of communication, such as facilitated communication. Because we live in southern California, it was easier to find participants who lived in California. However, we did include one participant with ASD and four supporters who lived outside of California.

All of the participants with ASD, as well as the majority of support participants, were Caucasian and middle-class. Again, this is a limitation on the ability to generalize the findings to other races, cultures, and socio-economic levels. It also raises the question of whether their relatively privileged status, in terms of resources and social capital, has been a determining factor in their academic success. This question was not addressed in this study and it certainly deserves attention in future research.

Another possible limitation was that prior to this study we shared personal relationships with one of the participants with ASD and three of her support providers. This brought both negative and positive aspects to the work. On the negative side, we may have assumed too much about these participants prior to data collection and, therefore, may not have been sufficiently open-minded. On the positive side, we did have background knowledge of the four participants. Therefore, we were able to spend more time on questions that focused on support and relationships and less time seeking background experiences of these individuals.

Although the findings of this study cannot be generalized to all individuals with ASD, it does have implications for many people with autism and raises important questions. For example, some might suggest that individuals who do not communicate either orally or through typing do not have relationships. An alternative possibility is that we have yet to find augmentative and alternative communication options that might allow them to communicate about their relationships. There is very little data to shed light on this issue. Anecdotally, however, many non-verbal people with ASD have been known to develop close and lengthy relationships. For example, Sue, a participant with autism in this study, began using AAC because of her friend who had been her psychologist at a younger age. This psychologist also had maintained a 20-year relationship with a non-verbal person who, in his late 20’s, began communicating for the first time using AAC. This psychologist was invited to be with this young man when he was first offered the opportunity to type with support. She was impressed with what she saw and decided to try this communication option with Sue. She made this decision based of her long-term relationship with Sue who, until that point, had never given any indication that she could use language to communicate. Additionally, Peyton, another participant with ASD in this study, maintains a deep and close friendship with a girl from her neighborhood with whom she grew up with. They were friends for many years prior to Peyton’s finding a reliable method of communication. These anecdotes suggest that social relationships between verbal and non-verbal individuals are possible. Clearly, the factors that enhance or discourage the development and maintenance of social relationships within the lives of non-verbal individuals with autism is an area which deserves greater attention from the research community.

Collecting data from participants who used AAC as their primary means of communication was challenging for both the researchers and the participants. Answering interview questions required a huge time commitment from the participants with ASD, their facilitators, and me. Often, four hours of interviewing would only yield a few pages of transcripts, leaving all involved, particularly the individual with ASD, exhausted. At first we found this frustrating, especially when traveling was involved. However, participants who used AAC were more than willing to receive questions beforehand or following an interview and work on questions independently with the help of their facilitator. This allowed our interview time to be used for further probing and clarification. The dedication and willingness of participants and their supporters to spend a great deal of time and energy providing me with data was a gift.

AAC posed an additional challenge because interviews required a support person to be present. This meant that interview sessions were not as private as we would have liked. However, each participant with ASD said they felt comfortable discussing these relationships openly with all of their support participants identified for this study.

We also faced challenges when interviewing individuals with ASD who used speech as their primary means of communication. As noted, we found that these individuals had particular problems when answering questions about how they felt about their supporters. One participant commented that this was hard for him because no one had ever asked him these types of question before. Therefore, we adapted interview questions and used vignettes to probe for responses. Our concern was that we might vary the questions and styles of questions too much, thus affecting the kind of data we were collecting. However, we felt all these accommodations were necessary.

One limitation during data collection was that we were not able to observe all the dyads in person. We were able to observe 6 of the 17 dyads in person and 4 dyads through pre-recorded videos and documentaries. We were not able to observe seven dyads for various reasons, including physical distance between participants and because some participants no longer interact together on a regular basis.

Qualitative studies often face limitations in regard to subjectivity, trustworthiness, and generalizability. As constructivist researchers, our subjective experience did influence the way the data was interpreted and presented. However, by recognizing this at the beginning of the study, we incorporated measures early on that allowed me to monitor subjectivity throughout the research process. Strategies mentioned in the methods section, such as my use of a researcher journal, ensured that our subjectivity was monitored. Issues regarding the trustworthiness of the findings and the generalizability of the substantive grounded theory, as well as steps taken to strengthen these aspects, were discussed in great in the methods section.

### 4.4. Implications

All participants with ASD identified trusting, unified, and supportive relationships as a key factor of their success. None of the participants, either supporters or individuals with ASD identified typical professional interventions (e.g., behavioral interventions) as key to their success or development. The findings of this present study call practitioners to rethink the current focus regarding education, services, and supports for individuals with autism. Unfortunately, the field does not seem to be headed towards a focus on supporting individuals with ASD through relationships. In fact, comprehensive programs based on professional interventions are growing in number and popularity. Within many of these traditional programs and the professional literature, support providers are encouraged to maintain a professional distance between themselves and the individuals they work with [20]. For example, most behavioral literature [59] only recognizes building “rapport” as a “precursor or warm-up strategy for establishing effective interventions” [20] (p. 226). However, for the participants with ASD in this study, the maintenance of a trusting and unified relationships were essential for effective support.

The findings of this study are similar to what Bordin [60,61] described as the “working alliance”. Bordin’s theory, based on his work in counseling and psychoanalysis, recognizes that effective interventions are wholly dependent on the quality of the bond that partners share. Effective bonds center around mutual feelings of liking, caring, and trust. Techniques and strategies alone do not matter. Rather, the focus should be on building and maintaining relationships. Bordin’s theory, as well as the findings of this study, has significant implications for supporting individuals with ASD. Perhaps the field has overlooked the potential importance of relationships in providing support to individuals with ASD and other disabilities.

Practitioners, parents, and anyone who cares about individuals with ASD, can provide support in the context of personal relationships. Participants in this study demonstrated that effective support required both beliefs and actions. Therefore, the first step towards supporting individuals with ASD through relationships is to examine one’s beliefs and assumptions regarding ASD. Once one understands what their assumptions are and what they are based on, one can begin to rethink them and examine the implications these assumptions have on others, specifically individuals with ASD.

The assumption that individuals with ASD are competent human beings capable of developing and maintaining personal relationships that might be supportive has no dangerous effects, because if wrong, no one would be hurt. However, if one assumes that individuals with autism are not capable of developing and maintaining personal relationships, and if that assumption is wrong, one would be doing that individual a great disservice. This thinking is based on what Donnellan [62] referred to as the “Criterion of the Least Dangerous Assumption”. When we cannot be certain, because we are not completely confident in what we know or have too little information, we should base our efforts, views, and perspectives on assumptions which, if wrong, will have the least dangerous effect on outcomes [62,63]. In sum, the least dangerous assumption is that though individuals with autism do have many challenges in social interaction, it is not necessary to infer that they are unwilling to be social and participating members of society.

## 5. Conclusions

This study has provided specific examples of how personal relationships between people with ASD and other individuals can develop and provide a major source of support for both members of the dyad. Supportive relationships involve: (1) a constant level of trust between both members of the dyad. Violations of trust may affect current and future relationships and attempts at support; (2) an intimate connection. Both members must have a deep and intimate understanding of each other. This concept is similar to what Kliewer and Biklen [21] termed “local understanding”; (3) mutual sharing of beliefs, emotions, desires, and goals, as well as mutual affection and support; (4) reciprocity between the members of the dyad. However, what is given and received does not have to be similar or equal (see also [46]); (5) varied patterns of relationship development; (6) members who presume that the other person is competent. This involves recognizing the person as an intelligent person who possesses all the characteristics of “personhood” (see also [17,64]); (7) members who see beyond labels and stereotypes (see also [50]); (8) sharing a vision of independence for the labeled individual. This involves ensuring that individuals have control over the decisions that affect their lives. Relationships are not based on the supporter’s power, control, or authority; (9) supporting the labeled individual’s effort to communicate; and (10) support within inclusive environments, as well, support is most effective when it is collaborative, consistent, and flexible.

Although these findings can be used as guides for supporting individuals with ASD through relationships, both support and relationships must be personalized. Therefore, relationships and supports should develop for individuals within their own life contexts. This last point is very important, yet often forgotten: a relationship is a two-way street. One should not assume that just because they may be open to a relationship with an individual with ASD, or any person with a disability, that the individual with a disability is necessarily interested in developing a relationship with them. As Norman Kunc, an individual with cerebral palsy, stated: “Do not try to be my friend. I deserve more than that. Get to know me. We may become friends” [65].

The goal of this study was to create a substantive grounded theory to further our understanding of supportive relationships in the lives of individuals with autism. The goal was not to develop a theory that would answer all questions about these relationships. Rather, the goal was to generate more questions. This study served as a preliminary analysis of a complex and virtually unexplored topic. Research should continue to explore how individuals with autism find support within personal relationships. There are numerous questions that warrant future exploration. For example, for individuals with ASD who are academically successful, what other factors have influenced their success? Possible factors to explore include race, sex, socio-economic status, intelligence, educational background of parents, or family status (i.e., single parent home, two-parent home). It would also be fruitful to explore supportive relationships in the lives of individuals with ASD at various times throughout their lives. At what point in life are these individuals most likely to develop relationships that provide support? At what point are they least likely? What factors limit or assist the development of these relationships? How do relationships that are not supportive affect these individuals? The questions to explore are almost limitless. Although this study did include observations, further studies could observe dyads closely for longer periods of time. For example, by focusing on only a few dyads, a researcher could more deeply explore the properties of the relationship. Future research must include the experiences and perspectives of both members of the dyad in order to fully understand these relationships.

## Figures and Tables

**Table 1:** Description of Participants.

Participant	Description	Sex	Race	Age (in Years)
**Sue Rubin**	Individual with Autism	Female	Caucasian	27
Rita	Mother	Female	Caucasian	50–59
Emily	Support Staff	Female	Caucasian	20–29
Aishling	Former Support Staff	Female	Middle Eastern	20–29
Lisanne	Former Support Staff	Female	Latina	30–39
**Tyler Fihe**	Individual with Autism	Male	Caucasian	19
Lynn	Mother	Female	Caucasian	50–59
Janna	Support Staff	Female	Caucasian	40–49
**Stephen Hinkle**	Individual with Autism	Male	Caucasian	26
Liz	Mother	Female	Caucasian	50–59
Claire	Former Support Staff	Female	Caucasian	30–39
Deborah	Educational Consultant	Female	Caucasian	50–59
**Peyton Goddard**	Individual with Autism	Female	Caucasian	31
Dianne	Mother	Female	Caucasian	60–69
Pat	Father	Male	Caucasian	60–69
Martha	Friend (Support Staff for 5 days)	Female	Caucasian	50–59
Mary	Support Staff	Female	Caucasian	40–49
**Matthew Ward**	Individual with Autism	Male	Caucasian	27
Nancy	Mother	Female	Caucasian	50–59
Tom	Stepfather	Male	Caucasian	50–59
Abby	Former Support Staff	Female	Caucasian	20–29
Sarah	Support Staff	Female	Caucasian	30–39

**Table 2:** Sources of Data.

Participant	Data Sources
**Sue Rubin**	Face-to-face interviews
	Observations
	Email correspondence
	Published documents
	Documentary-Autism is a World
	Additional public broadcasts
Rita	Face-to-face interview
	Published article
	Scenes in documentaries
Aishling	Face-to-face interview
	Scenes in documentary
Lisanne	Face-to-face interview
	Scenes in documentary
Emily	Face-to-face interview
	Email correspondence
	In-person observation with Sue
**Tyler Fihe**	Face-to-face interviews
	Observations
	Email correspondence
	Published documents Published video recording-Voices of Vision
Lynn	Face-to-face interview
	Scenes in video
Janna	Face-to-face interview
	Phone interview
	Scenes in video
	In-person observations
**Stephen Hinkle**	Face-to-face interviews
	Observations
	Email correspondence
	Presentation handouts
Liz	Face-to-face interview
	In-person observation
Deborah	Face-to-face interview
Claire	Face-to-face interview
**Peyton Goddard**	Face-to-face interviews
	Observations
	Email correspondence
	Published documents
	Additional documents
	Documentary–Helium Hearts
Dianne	Face-to-face interviews
	Scenes in documentary
	In-person observation
Pat	Face-to-face interviews
	Scenes in documentary
	In-person observation
Mary	Face-to-face interview
	In-person observation
Martha	Phone interview
**Matthew Ward**	Phone interview
	Documents
	Video recordings–Autism Project, University of Madison-Wisconsin
	Conference presentation transcript
Nancy	Phone interview
	Scenes in video
Tom	Phone interview
Abby	Phone interview
Sarah	Questionnaire
	E-mail correspondence

**Table 3:** Stages of Analysis.

Stage One	Stage Two	Stage Three
1. Analysis of data from separate participant categories within one group. (e.g., person with ASD, family supporters, non-family supporters).	1. Analysis of data from participant categories within all groups (e.g., all persons with ASD, all family supporters, all non-family supporters).	1. Analysis of data from all groups and all participant categories.
2. Analysis of data together as one group.		
3. Repeat for all groups.		

**Table 4:** Dimensions of Trustworthiness.

Recommendations	Actions of Researchers
Talk little, listen a lot	Encouraged participants to lead discussion
	Allowed conversations to flow
	Avoided interruptions
	Redirected through gestures and questions
	Expressed interest and ignorance to encourage participants to tell their own story
Record accurately	Effective systems of data collection were put into place
Begin writing early	Memo writing occurred throughout data collection and analysis
Let readers “see” for themselves	Theoretical sampling
Report fully	Multiple methods of data collection techniques and sources of data
Be candid	Researcher journal allowed us to examine our own subjectivity
Seek feedback	Peer debriefing and member checks
Try to achieve balance	Data analysis took place during data collection
Write accurately	Vignettes and quotes from participants were included

**Table 5:** Properties of Supportive Relationships.

Trust	Unity	Support
Developing Trust Testing for Trust	Staff and Friend Supporters/Relationships Family Supporters/Relationships Reciprocity - Intrigue and Uniqueness - Friendship - Affection and Love - Sensing Emotions - Spirituality - Influence and Advice - Learning and Growth - Providing a Focus - Inspiration - Pride	Shared Vision of Independence Presuming Competence Understanding Inclusion Communication Collaboration Consistency and Flexibility Personal Characteristics and Interaction Styles

## Appendix A. Interview Guides

Interview Guide–Participant with Autism

**Support in general**

1. Can you describe the support system that you have in place now? Who is involved and how are they involved?

2. What roles do you see each of these people playing?

3. How has the way you are supported changed from they way you were supported when you were younger?

4. Do you have any requirements for the people who support you? If so, what are they? Do they have to have a background in autism or supporting people with autism?

5. How do you think you are best supported? Can you give me examples? Can you give me an example of a time when someone was trying to support you but it was not successful?

6. How do changes in support affect you?

7. What do you do when you think you are not being successfully supported? How do you let them know?

8. How do you communicate with the people who support you? How do you negotiate support or things about support?

9. How important is the relationship you have with people who support you? How do you develop trust with someone who supports you?

10. Can you think of 2 or 3 people who have provided you support in your life? Would I be able to interview any of them? Could we talk about these relationships more specifically?

**Specific support relationships (Questions will be asked for each individual identified as a support)**

1. How do you describe the relationship you have with_________?

2. What do you think the goal of this relationship is? Do you think that it is the same goal that the person who supports you has? How are you accommodated or supported toward this goal? How do you measure the outcome of that goal?

3. How effective is this relationship in providing you with support? How do you think this person could be more effective in supporting you? Is the effectiveness of the support something you communicate about with the person who supports you?

4. How do you think the person who supports you perceives of the way they support you? Do you think your perspective of the support is similar to theirs?

5. Describe how you communicate with this support person? How do you think that affects the relationship and support?

6. Can you give me an example of a time a negotiation took place between you and the person who supports you? If negotiations do not take place, how are decisions about support decided upon?

7. What are the positive aspects of this relationship? What are the challenges?

Interview Guide–Supporter

**Support**

When and how did you first meet _________?

Can you describe how you established a relationship with ________? What were your first impressions of ________?

Can you give an examples of how you support _________?

What do you think the goal is of your relationship? Do you think that is the same goal the person you support has? How you support this person towards that goal?

How do you measure the outcome of that goal?

What are some of your personal characteristics that are help you support this person?

**Communication**

Describe how you and _________ communicate? How do you think that affects the relationship? How do you think that affects how you provide support?

Can you give me an example of a time a negotiation took place between you and _______? If no negotiations take place, how are decisions decided upon?

**Relationship**

How has your relationship changed since it was first established? How has support changed?

What are the positive aspects of the relationship? Can you give me an example?

What are the challenging aspects of the relationship? Can you give me an example?

What aspects of the relationship and support do you think needs work? What aspects would you like to maintain?

What are your concerns, hopes and fears for the future?

Any other comments about supporting this person?
